# Bumetanide induces post-traumatic microglia–interneuron contact to promote neurogenesis and recovery

**DOI:** 10.1093/brain/awad132

**Published:** 2023-04-21

**Authors:** Marine Tessier, Marta Saez Garcia, Emmanuelle Goubert, Edith Blasco, Amandine Consumi, Benoit Dehapiot, Li Tian, Florence Molinari, Jerome Laurin, François Guillemot, Christian A Hübner, Christophe Pellegrino, Claudio Rivera

**Affiliations:** Aix Marseille Univ, INSERM, INMED, 13273 Marseille, France; Neuroscience Center, 00014 University of Helsinki, Helsinki, Finland; Aix Marseille Univ, INSERM, INMED, 13273 Marseille, France; Aix Marseille Univ, INSERM, INMED, 13273 Marseille, France; Aix Marseille Univ, INSERM, INMED, 13273 Marseille, France; Aix Marseille Univ, CNRS, IBDM-UMR7288, Turing Center for Living Systems, 13288 Marseille, France; Institute of Biomedicine and Translational Medicine, University of Tartu, 50411 Tartu, Estonia; Aix Marseille Univ, INSERM, MMG, 50411 Marseille, France; Aix Marseille Univ, INSERM, INMED, 13273 Marseille, France; The Francis Crick Institute, London NW1 1AT, UK; Institut für Humangenetik, Universitätsklinikum Jena, 07747 Jena, Germany; Aix Marseille Univ, INSERM, INMED, 13273 Marseille, France; Aix Marseille Univ, INSERM, INMED, 13273 Marseille, France; Neuroscience Center, 00014 University of Helsinki, Helsinki, Finland

**Keywords:** traumatic brain injury, microglia, chloride homeostasis, neuroinflammation, GABAergic transmission

## Abstract

Although the Na-K-Cl cotransporter (NKCC1) inhibitor bumetanide has prominent positive effects on the pathophysiology of many neurological disorders, the mechanism of action is obscure. Attention paid to elucidating the role of Nkcc1 has mainly been focused on neurons, but recent single cell mRNA sequencing analysis has demonstrated that the major cellular populations expressing *NKCC1* in the cortex are non-neuronal.

We used a combination of conditional transgenic animals, *in vivo* electrophysiology, two-photon imaging, cognitive behavioural tests and flow cytometry to investigate the role of Nkcc1 inhibition by bumetanide in a mouse model of controlled cortical impact (CCI).

Here, we found that bumetanide rescues parvalbumin-positive interneurons by increasing interneuron-microglia contacts shortly after injury. The longitudinal phenotypic changes in microglia were significantly modified by bumetanide, including an increase in the expression of microglial-derived BDNF. These effects were accompanied by the prevention of CCI-induced decrease in hippocampal neurogenesis. Treatment with bumetanide during the first week post-CCI resulted in significant recovery of working and episodic memory as well as changes in theta band oscillations 1 month later.

These results disclose a novel mechanism for the neuroprotective action of bumetanide mediated by an acceleration of microglial activation dynamics that leads to an increase in parvalbumin interneuron survival following CCI, possibly resulting from increased microglial BDNF expression and contact with interneurons. Salvage of interneurons may normalize ambient GABA, resulting in the preservation of adult neurogenesis processes as well as contributing to bumetanide-mediated improvement of cognitive performance.

## Teaser

Tessier *et al*. identify a mechanism underlying the neuroprotective effect of bumetanide in a controlled cortical impact model. Bumetanide rescues parvalbumin-positive interneurons by increasing interneuron-microglia contacts shortly after injury. Interneuron rescue in turn normalizes ambient GABA, resulting in the preservation of adult neurogenesis.

## Introduction

Traumatic brain injury (TBI) is one of the most prevalent pathologies worldwide, with more than 20 million people affected each year.^1^ However, possibilities for intervention to prevent comorbidities and long-lasting sequelae that frequently follow TBI are limited. Understanding the changes and key players involved in the pathophysiology of the latent phase may help to prevent or reduce some of the comorbidities that accompany TBI.

TBI can be frequently associated with complications such as epileptic seizures, depression and memory impairment.^2^ This may partly result from the alteration of hippocampal information processing and neurogenesis.^3–5^ However, the mechanisms regulating neurogenesis following TBI remain elusive because of contradictory reports.^3,6^

Adult neurogenesis is controlled by local GABAergic interneurons and GABA_A_ receptors. In particular, the release of GABA by parvalbumin (PV)-expressing interneurons controls the quiescent state of radial glia-like cells (RGL) in the dentate gyrus (DG).^7^ Studies in humans and mouse models of TBI have demonstrated a progressive loss of PV-expressing interneurons in both the ipsi- and the contralesional hippocampus.^8–10^ We have previously shown that the inhibition of chloride uptake by bumetanide, an inhibitor of Na-K-Cl (NKCC1) cotransport, significantly decreased PV interneuron loss with a positive effect on both depressive-like behaviour and cognitive performance.^6^ Despite the potential clinical relevance of these results, the underlying mechanisms remain unknown.

Previous studies on chloride uptake in the brain mainly focused on neuron-intrinsic mechanisms, although the expression of the principal chloride cellular uptake transporter Nkcc1 is mainly expressed in non-neuronal cells.^11^

Glial cells are involved early on in the temporal sequence of the neuroinflammatory processes following TBI.^12^ Indeed, quiescent glial cells are rapidly activated by a process called ‘reactive gliosis’. The recruitment of peripheral neutrophils is also observed, which is followed by infiltration of lymphocytes and monocyte-derived macrophages. Simultaneously, the release of pro-inflammatory and anti-inflammatory cytokines promotes and/or inhibits the post-traumatic neuroinflammatory response.^13^ Activated microglia trigger and maintain astrocytic activation through the release of cytokines, which in turn act on surrounding glial cells and neurons.^14^ In addition, recent reports showed an important pro-survival function of physical contact between activated microglia and interneurons, especially PV-positive interneurons.^15^

In this study, we identified a novel mechanism involving cellular chloride uptake in microglia, interneuron survival and neurogenesis that is linked to TBI-induced cognitive decline.

## Material and methods

For full description of experimental procedures please see the Supplementary material.

### Experimental animals

Wild-type mice had a C57bl6-J background. Three transgenic lines were used, Nestin-GFP,^16^ hGFAP-Cre^17^ (line 77.6 mice Stock No. 024098; The Jackson Laboratory) × Nkcc1 flox^18^ and B6.129P-Cx3cr1tm1Litt/j,^19^ and maintained on a mixed genetic background. All animal experiments complied with the French and Finnish ethical committees, which approved all experimental procedures (No: APAFIS#2797 and ESAVI/3183/2022), and the ARRIVE guidelines and were carried out in accordance with the UK Animals (Scientific Procedures) Act, 1986 and associated guidelines and EU Directive 2010/63/EU for animal experiments.

### Controlled cortical impact model

Buprenorphine (0.03 mg/kg) was injected intraperitoneally into 10-week-old C57bl6-J males 30 min before surgery. Mice were then anaesthetized using 4% isoflurane mixed with air and enriched with oxygen (0.3%) and positioned in a stereotaxic frame (David Kopf Instruments). Body temperature was monitored throughout the procedure using a rectal probe and maintained at 37 ± 2°C with a heating pad (Harvard Apparatus). A unilateral craniotomy was performed at the level of the right posterior parietal cortex. Controlled cortical impact (CCI) was performed using a Leica impactor with standardized parameters (tip diameter 3 mm, 6 m/s speed, 1.5 mm depth and 200 ms duration). Animals were allowed to recover on the heating pad before being transferred to a post-surgical room. Before the experiment, animals were randomly assigned to one of the following groups: sham-vehicle (Sham Veh), sham-bumetanide (Sham Bum), CCI-vehicle (CCI Veh) or CCI-bumetanide (CCI Bum).

### Drug delivery

A bumetanide stock solution 20 mM (Sigma-Aldrich, B3023) was prepared by dissolving 36.4 mg of powder into 1 ml of absolute ethanol. Intraperitoneal injections were performed twice daily 12 h apart either during the first week post-CCI at 2 mg/kg or during the 1-week preceding CCI.

Minocycline (Sigma-Aldrich, M9511) was injected twice daily, 12 h apart (45 mg/kg) for 1 week. The vehicle solution consisted of the same preparation but lacked the bumetanide/minocycline powder to respect volume and diluent.

### Immunohistochemistry

Immunohistochemistry was performed on 4% paraformaldehyde fixed free-floating sections. Incubation with primary antibodies diluted in PBS with 5% NGS and 0.3% Triton X-100 was carried out at 4°C overnight using rabbit anti-doublecortin (ab18723, Abcam, 1:1000), mouse anti-PV (p3088, Sigma, 1:500), rabbit anti-Iba1 (W1W019-19741, Wako, 1:500), mouse anti-GFAP (MAB360, Merck Millipore, 1:500), mouse anti-BDNF antibody (MAB#918), mouse anti-RFP antibody (MA5-15257, ThermoFisher Scientific, 1:500), chicken anti-GFP antibody (AB_2307313, Avès Labs, 1:300) as well as corresponding Alexa Fluor-conjugated secondary antibodies (ThermoFisher Scientific, 1:500).

### Parvalbumin microglia contact quantification

Confocal image stacks with double immunofluorescent labelling (PV and Iba1) were acquired using a LSM-800 Zeiss confocal microscope Plan Fluor 40×/1.30 NA oil immersion objective. Optical sections were collected from the ipsi- and contralesional hippocampi of mice treated and non-treated with bumetanide at 3 and 7 days post-CCI. Images were then reconstructed in 3D with Imaris Software. Then, contacts between microglia and PV interneurons were quantified with ImageJ plugin SynapCountJ.

### Flow cytometry analysis

Fresh cortical tissues were dissected and gently chopped and minced in 1 ml ice-cold flow cytometry staining buffer (PBS + 1% foetal calf serum) through a 100 μm filter on ice. The homogenates were first blocked in PBS with 10% rat serum with gentle rotation in a cold room and then stained for 1 h protected from light at 4°C with CD206-FITC (Cat. No. 141704, BioLegend) + MHCII-PE (Cat. No. 107608, BioLegend) + CD11b-PerCP/Cy5.5 (Cat. No. 101228, BioLegend) + CD45-APC (Cat. No. 103112, BioLegend). After staining, cells were fixated with 8% PFA for 30 min and centrifuged at 2000 rpm. Samples were acquired with a 2-laser, 6-colour cytometer (Gallios, Beckman Coulter). The gating strategy was determined based on the specific staining of markers for microglial populations.^20^

### Live imaging

Imaging was performed on 5-month-old male mice (B6.129P-Cx3cr1tm1Litt/j, *n* = 5) carrying a cranial window surrounded by a metallic head plate. One month after surgery, trained awake animals were head-fixed on a Mobile HomeCage platform (MHC V5, Neurotar) under a custom-built two-photon microscope (Femtonics) equipped with a Mai Tai Ti:Sapphire Ultrafast Laser (Spectra-Physics) and imaged longitudinally using a Nikon Apo LWD 25× objective with 1.10 NA for 5 days. An image stack with a 5 mm step size covering 300 mm was acquired. After a 30 min baseline recording, injury was then achieved by focal excitation of one cell for 20 s at 75 μs/pixel at 100% laser intensity. After the injury, animals were treated (Vehicle/Bumetanide) twice per day and imaged, and *z*-stacks were collected at 5 min intervals for 1 h.

To compute the microglial cell dynamics, we designed a semi-automated procedure using a using Python. Subvolumes were cropped around each cell before undergoing spatial registrations using pystackreg (G. Lichtner, pystackreg, 2022), https://github.com/glichtner/pystackreg). Cell dynamics were evaluated from the segmented image by calculating the percentage of binary pixels changing in value from one time point to another.^21^

### Behavioural tests

One month post-CCI, mice were tested on different tasks using the object recognition paradigm to test the individual components of episodic-like memory, namely the novel object recognition (NOR) task and the object displacement task (ODT).^22^ For NOR, mice were placed in the centre of a box (Noldus Apparatus, 38.5 cm × 38.5 cm) and allowed to explore the space freely for 10 min; then, two identical objects were added. After a 3 min retention time, one of the objects was replaced, and the time of exploration was measured for a 3 min period. The same experiment was also performed with a 1 h retention time. A similar protocol was used for the ODT, with the difference that after the 3 min retention time, one of the objects was moved, and the time of exploration of each object was recorded.

Mice were also tested in the Barnes maze test. One month post-CCI, mice were trained twice daily for 4 days. On trial probe day (Day 5), the target box was replaced with a false escape. The distance travelled, the number of errors, the time spent in the target zone and the latency to reach the target hole were measured.

Finally, mice were tested for anxiety using the elevated plus maze (EPM) test (Ugo Basile Co.). For each test, mice were placed in the centre square, facing an open arm and allowed to move freely for 5 min. Entries and time spent in one of the two open or closed arms and centre were monitored. Recording and analyses were done using Ethovision software (Noldus).

### *In vivo* electrophysiological recordings

Scalp EEG recordings were performed in freely moving mice 3 weeks post-CCI. Telemetric recording electrodes were implanted at the level of the posterior parietal cortex (2 mm posterior to and 1.5 mm left of the bregma). A reference electrode was placed rostral in the cerebellum. After a 72-h recovery, EEG (amplified 31 000, filtered at 0.1–120 Hz pass, acquired at 1000 Hz) was monitored using a telemetric system (Emka Technologies S.A.S) for 3 days, 24 h per day.

Multisite intrahippocampal recordings were performed by implanting a one-shank 16-channel linear probe (A1 × 16–3 mm-100–177, Neuronexus) through a 1 mm diameter craniotomy at the posterior parietal level (2 mm posterior, 1.5 mm left of the bregma and 1 mm ventral) on head-restrained animals trained on a Mobile HomeCage (MHC V5, Neurotar). Animals were recorded three times for 10 min. Recordings were acquired using Allego software (SmartBox Pro™, Neuronexus) and a sampling rate of 30 K Hz per channel.

For EEG recordings, animal temperature as well as movement were used to determine active and non-active time windows. Delta (1–4 Hz), theta (4–12 Hz) and gamma (30–80 Hz) bands^23^ were normalized using the ratio of power in the first to the last minute within each recording session. The mean normalized power of active time windows was subtracted from non-active for each recording.

From intrahippocampal recordings, individual units were isolated from multisite LFP recordings using Spyking-Circus.^24^ Single-unit activities were further classified into wide-spiking (WS) and narrow-spiking (NS) units^25^ based on the spike waveform, autocorrelogram and cross-correlogram. NS and WS presumably corresponded to putative inhibitory and excitatory units, respectively (Supplementary Fig. 4).^26,27^

### Statistical analysis

All mean values are given with the standard error of the mean (SEM). Normality was tested using a D’Agostino–Pearson test. Two-tailed Student's *t*-tests were used for testing statistical significance between two groups while Mann–Whitney tests were used when the normality test failed. Ordinary one-way ANOVA tests were used to compare three or more groups. In the event that the normality assumption was violated here, the Kruskal–Wallis test was used. When variances were unequal, the Brown–Forsythe ANOVA was used.

Statistical analyses were carried out using Prism Version 8.3.0 (GraphPad software, Inc., La Jolla, CA, USA) and are represented as follows: *P < 0.05, **P < 0.01 and ***P < 0.001. All results and statistical significance are reported in Supplementary Table 1.

## Results

### Bumetanide ameliorates CCI-induced memory impairment and pathological neuronal activity

**Figure 1:**
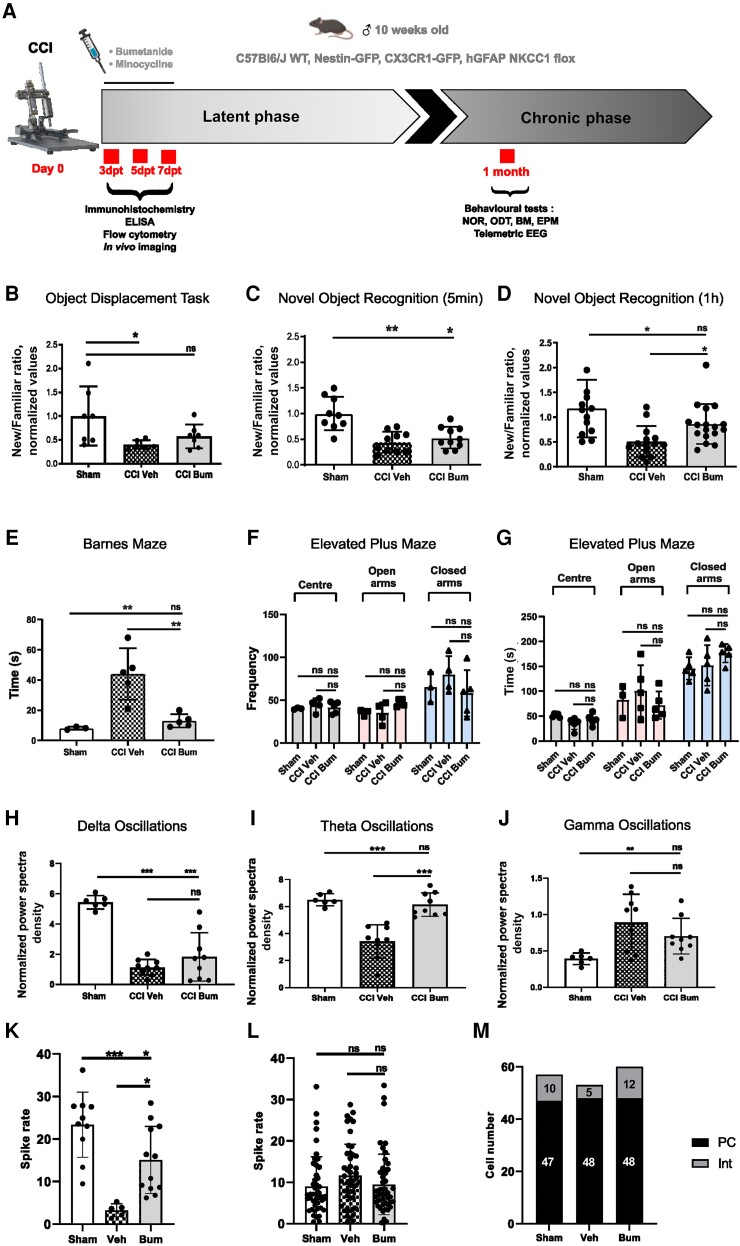
**Effect of bumetanide on CCI-induced behavioural changes**. (**A**) Schematic representation of the experimental timeline. (**B**) The object displacement task (ODT) after a 5 min retention time. (**C**) The novel object recognition (NOR) task after a 1-h retention time. (**D**) The NOR task after a 5-min retention time. For all experiments, the results are presented as a ratio of time of new versus familiar. *n* = 10, 11 and 10, respectively. (**E**) Time spent finding the target box in the Barnes maze (BM). (**F**) Number of entries into different zones of the elevated plus maze (EPM). (**G**) Time spent in different zones of the EPM. *n* = 3, 5 and 5, respectively. (**H**) Normalized spectral density of delta band in mice 1-month post-controlled cortical impact (CCI). (**I**) Normalized spectral density of theta band. (**J**) Normalized spectral density of gamma band. *n* = 6, 9 and 9, respectively. (**K**) Spike rate of narrow-spiking (NS) units. (**L**) Spiking rate of wide-spiking (WS) units. (**M**) Total number of isolated cells. Data in **B** were analysed using the Kruskal–Wallis test. Data in **C**–**G**, **L** and **M** were analysed using one-way ANOVA with Dunnett's *post hoc* test. Data in **H**–**J** were analysed using one-way ANOVA with Tukey’s *post hoc* test. *P < 0.05; **P < 0.01; ***P < 0.001. Bum = bumetanide; dpt = days post-testing.

To investigate the effects of bumetanide on post-TBI cognitive behaviour, we performed cognitive performance tests, the NOR task and the ODT. CCI induced a significant decrease in the recognition ratio of the tasks examined after normalization to sham conditions unless otherwise stated (ODT: CCI Veh 0.38 ± 0.06; NOR 5 mins: CCI Veh 0.44 ± 0.20; NOR 1 h Sham 1.17 ± 0.58 versus CCI Veh 0.507 ± 0.31; Fig. 1B–D). Interestingly, the strongest effect of bumetanide was found in NOR with 1 h retention (CCI Bum 0.86 ± 0.40; Fig. 1D). We then used a spatial memory test, the Barnes maze, which showed that CCI induced a significant increase of the latency in reaching the target hole (Sham 8.00 ± 0.58 versus CCI Veh 44.00 ± 7.61; Fig. 1E). Similarly, bumetanide treatment resulted in a better performance compared to vehicle (CCI Bum 13.00 ± 1.95; Fig. 1E). Finally, as NOR and the ODT are strongly influenced by anxiety, we used the EPM to test this parameter and did not find significant differences in the number of entries that animals made into each zone (Centre: Sham 40.00 ± 0.58 versus CCI Veh 44.00 ± 4.02 versus CCI Bum 41.40 ± 3.11; Open arms: Sham 35.33 ± 2.186 versus CCI Veh 35.00 ± 6.18 versus CCI Bum 46.00 ± 1.86; Closed arms: Sham 65.33 ± 10.11 versus CCI Veh 79.75 ± 10.82 versus CCI Bum 58.00 ± 12.03) (Fig. 1F). There were also no differences in the time that animals spent in the centre (Sham 51.18 ± 1.657 versus CCI Veh 35.00 ± 5.343 versus CCI Bum 43.048 ± 5.306), nor in the open arms (Sham 82.44 ± 18.79 versus CCI Veh 100.70 ± 23.10 versus CCI Bum 71.98 ± 12.39), nor in the closed arms (Sham 145.72 ± 9.97 versus CCI Veh 151.96 ± 18.27 versus CCI Bum 176.264 ± 8.27) (Fig. 1G). These results show that CCI had a negative impact on learning performance and spatial memory, and that it was not linked with anxiety.

Memory impairment following TBI are characterized by changes in theta rhythms.^28–30^ Using telemetric EEG, we found that at 1-month post-CCI, the power of theta band oscillations in vehicle-treated animals was significantly lower compared to sham (Sham 6.50% ± 0.18 versus CCI Veh 3.42% ± 0.41; Fig. 1I). Bumetanide treatment normalized the theta band power and was significantly different from vehicle-treated animals (CCI Bum 6.14% ± 0.29; Fig. 1I). The power of the delta band was lower in CCI animals and was not affected by bumetanide treatment (Sham 5.44% ± 0.18 versus CCI Veh 1.14% ± 0.17 versus CCI Bum 1.82% ± 0.53; Fig. 1H). Similarly, the power of the gamma band was significantly higher in vehicle-treated animals compared to sham but not changed in bumetanide animals (Sham 0.39% ± 0.03 versus CCI Veh 0.89% ± 0.13 versus CCI Bum 0.70% ± 0.08; Fig. 1J). To assess the impact on single-unit activity, we isolated putative interneuron and principal neuron single-units from intrahippocampal multisite recordings. Here we found that the spike rate of putative interneurons was significantly decreased in vehicle compared to sham animals (Sham 23.38 ± 2.42 Hz versus CCI Veh 3.26 ± 0.6824 Hz) but not in bumetanide animals (Bum 15.08 ± 2.274 Hz; Fig. 1K). Interestingly, the putative principal cell unit spike rate was not affected in all groups (Fig. 1L). The number of total recorded putative interneurons was much lower in vehicle- (5) than sham- (10) and bumetanide- (12) treated animals, whereas the principal cell number was constant in all conditions (Sham: 47 cells; Veh: 48 cells; Bum: 48 cells; Fig. 1M). These results suggest that bumetanide ameliorates CCI-induced changes in interneuron function.

### Bumetanide rescues CCI-induced changes in secondary neurogenesis and PV interneuron loss

**Figure 2:**
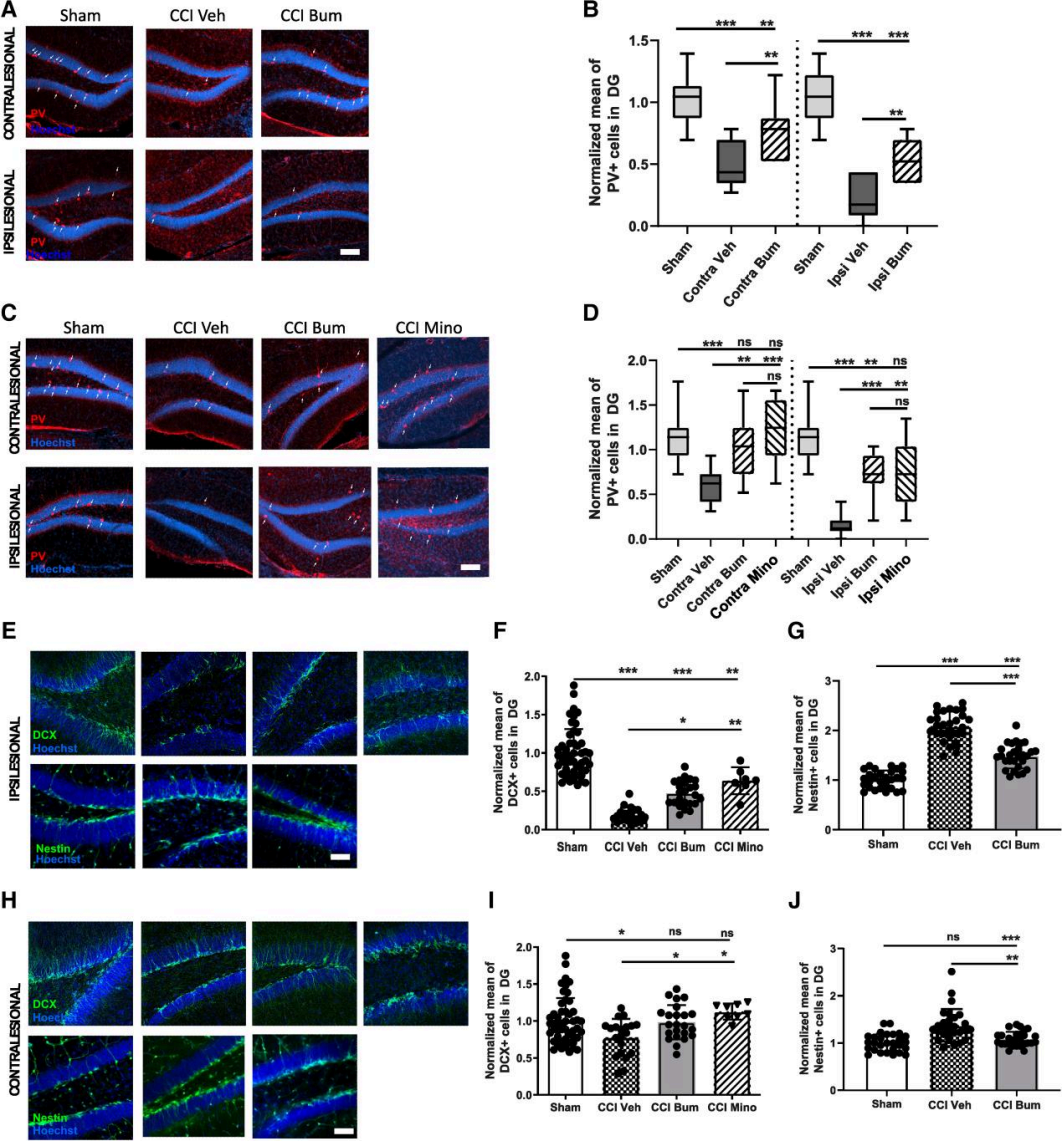
**Effect of bumetanide and minocycline on CCI-induced changes in adult neurogenesis and PV interneuronal loss in the DG**. (**A**) Parvalbumin (PV) labelling (white arrows) at 3 days post-controlled cortical impact (CCI) in the contralesional (*top*) and ipsilesional (*bottom*) dentate gyrus (DG) of sham-, CCI vehicle- and CCI bumetanide (Bum)-treated animals (Scale bar = 100 μm). (**B**) Quantification of PV-positive cells 3 days post-CCI in the contralesional and ipsilesional DG of sham-, CCI vehicle- and CCI bumetanide-treated animals. (**C**) As for **A** at 7 days post-CCI with minocycline (Mino)-treated animals added. (**D**) As for **B** at 7 days post-CCI. (**E**) DCX (*top*) and Nestin (*bottom*) labelling at 7 days post-CCI in the ipsilesional DG of sham-, CCI vehicle, bumetanide- and minocycline-treated animals (Scale bar = 100 μm). (**F**) Quantification of DCX-positive cells 7 days post-CCI in the ipsilesional DG of sham-, CCI vehicle-, bumetanide- and minocycline-treated animals. (**G**) Quantification of Nestin-positive cells 7 days post-CCI in the ipsilesional DG of sham-, CCI vehicle-, bumetanide- and minocycline-treated animals. (**H**) As for **E** in the contralesional DG. (**I**) As for **F** in the contralesional DG. (**J**) As for **G** in the contralesional DG. *n* = 7 animals per condition, three slices per animal. Data in **B**, **D**, **F**, **G** and **I** were analysed using one-way ANOVA with Dunnett's *post hoc* test. Data in **J** were analysed using a Kruskal–Wallis test. *P < 0.05; **P < 0.01; ***P < 0.001.

Interneurons are important contributors to brain oscillations.^31^ PV-positive interneurons are a particularly vulnerable subpopulation following CCI.^7^ In addition, the impact of CCI on interneuron activity and reduction in numbers suggest changes in the interneuron population of the hippocampus following CCI. We quantified PV-positive interneurons in the granular layer of the ipsi- and contralesional DG during the first post-traumatic week at 3 and 7 days post-CCI. Both sides showed a significant reduction in the number of PV interneurons at 3 days post-CCI (normalized value on sham: Sham Contra 1.00 ± 0.20 versus Contra Veh 0.52 ± 0.18 and Sham Ipsi 1.00 ± 0.22 versus Ipsi Veh 0.22 ± 0.18; Fig. 2A and B). This loss was significantly reduced by bumetanide treatment in the contra- (0.77 ± 0.23) and ipsilesional side (0.51 ± 0.1; Fig. 2A and B). We also found a significant reduction in the PV-containing interneurons survival at 7 days post-CCI in both sides (normalized value on sham: Sham Contra 1.0 ± 0.27 versus Contra Veh 0.59 ± 0.18 and Sham Ipsi 1.00 ± 0.27 versus Ipsi Veh 0.14 ± 0.12; Fig. 2C and D). This loss was significantly reduced at the contra- (1.02 ± 0.34) and ipsilesional sides (0.71 ± 0.24, Fig. 2C and D) by bumetanide treatment. Bumetanide-induced interneuron survival was not restricted to the DG, as we found a similar effect on other hippocampal regions (in ipsilesional CA3: Sham 1.00 ± 0.12 versus Ipsi Veh 0.52 ± 0.09; and in ipsilesional CA1: Sham 1.00 ± 0.25 versus Ipsi Veh 0.37 ± 0.07).

Ambient GABA, provided by the ongoing activity of DG interneurons and particularly PV interneurons, plays a role in the proliferation and migration of granular cell progenitors.^6,32^ A loss of PV interneurons may significantly contribute to the TBI-induced changes in stem cell proliferation by reducing GABA release. At 7 days post-CCI, we observed a significant reduction in the number of doublecortin (DCX)-positive neurons within both the ipsi- and contralesional DG (normalized value on sham: Sham Ipsi 1.00 ± 0.31 versus CCI Veh Ipsi 0.19 ± 0.09 and Sham Contra 1.00 ± 0.31 versus CCI Veh Contra 0.78 ± 0.24, Fig. 2E, F, H and I). This coincided with an increase in the number of Nestin-positive cells within the DG (Sham Ipsi 1.00 ± 0.17 versus CCI Veh Ipsi 2.08 ± 0.29 and Sham Contra 1.00 ± 0.18 versus CCI Veh Contra 1.37 ± 0.34; Fig. 2E, G, H and J). Bumetanide treatment reduced the number of Nestin-positive cells (CCI Bum Ipsi 1.4 ± 0.24 and CCI Bum Contra 1.0 ± 0.15; Fig. 2E, G, H and J) and triggered an increase in the number of DCX-positive cells (CCI Bum Ipsi 0.47 ± 0.16 and CCI Bum Contra 0.98 ± 0.23; Fig. 2E, F, H and I).

**Figure 3:**
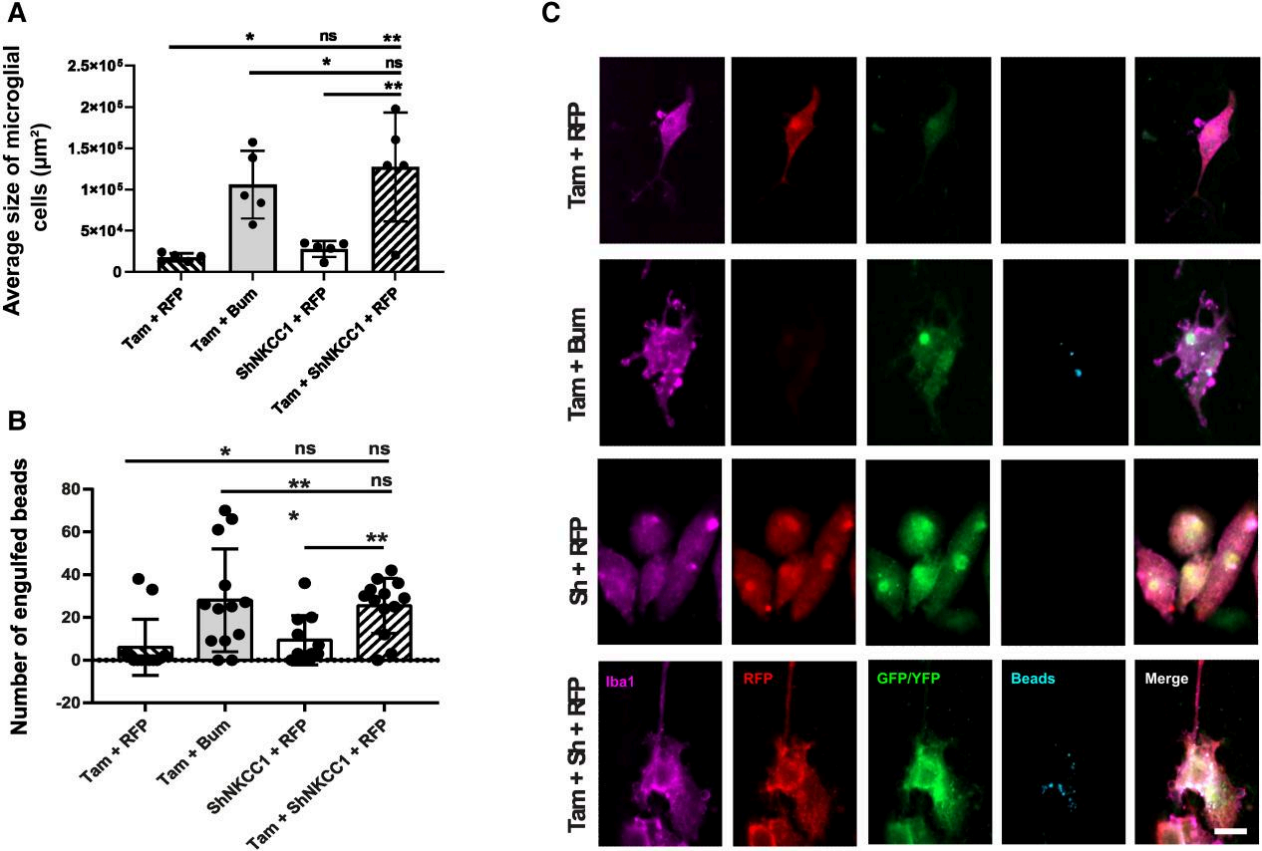
**Effect of impaired chloride uptake in microglia morphology and phagocytosis capacity**. (**A**) Average size of primary microglia cells from CX3CR1-Cre ERT2 mice under different conditions: co-transfected with pSico-sh Nkcc1 and pCAG-RFP, treated with bumetanide (Bum) and tamoxifen (Tam), transfected with pCAG-RFP and treated with Tam, co-transfected with pSico-sh Nkcc1 and pCAG-RFP and treated with Tam. *n* = 4 wells per condition and five cells chosen randomly per well. (**B**) Number of latex beads engulfed by cells in the different conditions. *n* = 4 wells per condition and 12 cells chosen randomly per well. (**C**) Immunostainings of the primary microglia cell culture from CX3CR1-Cre ERT2 mice under the different conditions. Anti-Iba1 was used to label microglial cells (purple), anti-RFP to label the pCAG-RFP plasmid (red) and GFP to enhance the pSico-sh Nkcc1 fluorescence (green). YFP was expressed when cells were treated with Tam (green) and the beads were labelled with a Rabbit IgG-DyLight 633 Complex (blue). All data were analysed using a one-way ANOVA with Dunnett's *post ho*c test. *P < 0.05; **P < 0.01; ***P < 0.001; ns = not significant.

### Bumetanide rescues CCI-induced changes in microglia phenotype and increases contact with PV interneurons

Neuroinflammation is present in the primary (acute) and secondary (chronic) stages of TBI,^12^ where microglia and astrocyte activation are involved in the mechanisms leading to both adverse and beneficial effects. Under physiological conditions, Nkcc1 is predominantly expressed in astrocytes and microglia compared to neurons.^33^ Therefore, we wondered whether these cell populations contribute to the effect of bumetanide. This was monitored in GFAP-Nkcc1-KO mice. Although we observed an abnormal morphology of astrocytes in this transgenic line, we did not find a significant increase in interneuron survival following CCI, suggesting that astrocytic chloride homeostasis is not relevant in CCI-induced apoptosis. The full description is provided in the Supplementary material and Supplementary Figs 1 and 2.

TBI is known to induce a strong inflammatory reaction.^34^ To see if TBI-induced effects on PV survival and proliferation are sensitive to microglial activation, we used the anti-inflammatory drug minocycline. Interestingly, treatment with this agent resulted in similar effects on both PV survival (Contra Veh 0.59 ± 0.18 versus Contra Mino 1.21 ± 0.34 and ipsilesional side Ipsi Veh 0.14 ± 0.12 versus Ipsi Mino 0.74 ± 0.39; Fig. 2A­­­­–D) and newborn neurons (CCI Mino Ipsi 0.63 ± 0.17 and CCI Mino Contra 1.12 ± 0.10; Fig. 2E–H). These results suggest that microglia activation affected post-CCI interneuron survival and adult born neuron production.

To investigate whether the effect of bumetanide depends on Nkcc1 in microglia, we used a transgenic approach for tamoxifen (Tam)-inducible knockdown of Nkcc1 in CX3CR1-positive microglia. Here, we observed that bumetanide treatment induced a significant increase in the average cell size in the control condition (Tam + Bum 105 732 μm^2^ ± 18 324 versus ShNKCC1 + RFP 27 650 μm^2^ ± 4322 versus Tam + RFP 17 482 μm^2^ ± 2164). Interestingly, knockdown of Nkcc1 also induced an increase in size (Tam + ShNKCC1 + RFP 127 090 μm^2^ ± 29 570 versus ShNkcc1 + RFP 27 650 μm^2^ ± 4322 versus Tam + RFP 17 482 μm^2^ ± 2164) (Fig. 3A and C). Similar results were found in BV2 cells (Supplementary Fig. 3). Then, we investigated the phagocytosis capacity of the cells in the different conditions. Bumetanide treatment induced a significant increase in the number of engulfed beads (Tam + Bum 28.00 ± 6.66 versus ShNkcc1 + RFP 9.34 ± 3.45 versus Tam + RFP 6.08 ± 3.64). Knockdown of Nkcc1 had similar effect (Tam + ShNkcc1 + RFP 25.46 ± 3.58 versus ShNkcc1 + RFP 9.34 ± 3.45 versus Tam + RFP 6.08 ± 3.64) (Fig. 3B and C). These results suggest that bumetanide regulated microglia morphology and activation by modulating Nkcc1 activity.

**Figure 4:**
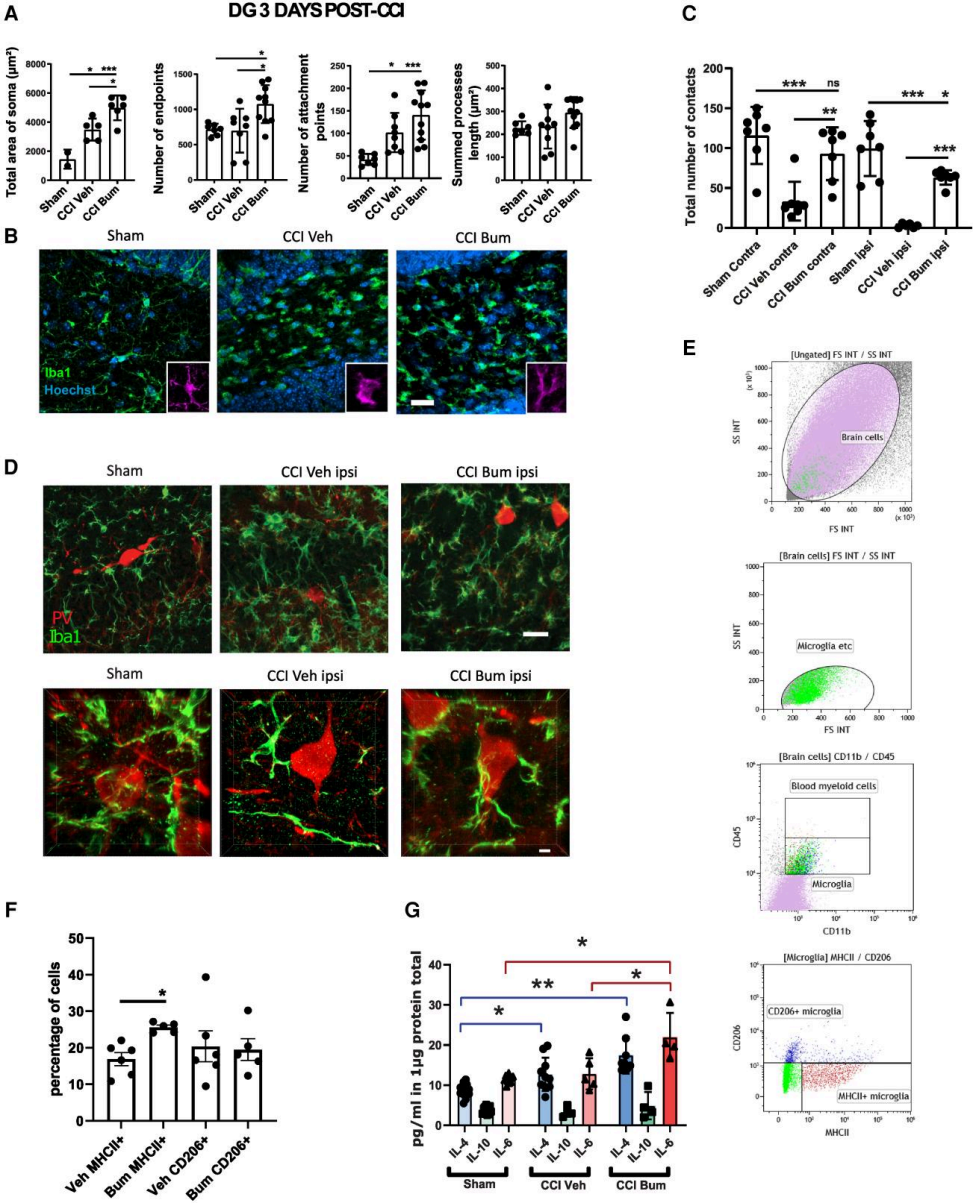
**Effect of bumetanide on microglia phenotypes and morphological changes after CCI in the DG 3 days post-CCI**. (**A**) Total area of Iba1+ cells soma, quantifications of Iba1+ process end points, attachment points and length in the ipsilesional DG. *n* = 5 animals, two slices per animal. (**B**) Iba1 immunostaining from sham, CCI-vehicle and CCI-bumetanide treated animals. (**C**) Quantification of the number of contacts between microglia (Iba1 staining) and PV interneurons at 3 days post-CCI. *n* = 5 animals per condition, three slices per animal. (**D**) Examples of PV and Iba1 immunostaining from sham-, CCI vehicle- and CCI bumetanide-treated animals in the ipsilesional DG, 3D representations (*top*) and stack (*bottom*). (**E**) Flow cytometry gating plots at 3 days post-CCI on the ipsilesional side. (**F**) Analysis of microglia phenotypes by detection of MHCII^+^ (pro-inflammatory) and CD206^+^ (pro-phagocytosis) markers by flow cytometry on the ipsilesional side at 3 days post-CCI. *n* = 5–6 animals per condition. (**G**) Quantity of IL-4, IL-10 and IL-6 produced in the ipsilesional hippocampus. Phenotype changes were analysed by *t*-test. Number of contacts and morphological analysis (quantified using the ImageJ plugins Neurphology and SynapCountJ) were analysed using one-way ANOVA with Dunnett's *post hoc* test. Interleukin production was analysed using a Brown–Forsythe ANOVA with Dunnett's *post hoc* test. *P < 0.05; **P < 0.01; ***P < 0.001; ns = not significant.

Furthermore, bumetanide treatment *in vivo* induced a significant increase of microglia end point numbers (Sham 715.9 ± 81.03 versus CCI Veh 698.8 ± 331.2 versus CCI Bum 1076.0 ± 85.13), number of attachment points (Sham 41.86 ± 12.40 versus CCI Veh 101.9 ± 43.70 versus CCI Bum 140.8 ± 54.96) and soma area (Sham 1458 ± 652.7 μm^2^ versus CCI Veh 3494 ± 757.6 μm^2^ versus CCI Bum 4985 ± 850.6 μm^2^) (Fig. 4A and B) in the ipsilesional DG 3 days post-CCI.

Microglia process motility and their interactions with interneurons play an important role in PV survival.^35^ We found significant differences in the number of contacts between microglial cells and PV interneurons between vehicle and bumetanide-treated animals at 3 days post-CCI in both the contra- (CCI Veh 40.00 ± 24.92 versus CCI Bum 103.50 ± 23.55) and ipsilesional side (CCI Veh 2.75 ± 2.48 versus CCI Bum 63.20 ± 9.04) (Fig. 4C and D). In addition, there was no significant difference between Sham and CCI Bum in the contralesional side (Sham Contra 115.9 ± 13.50 versus CCI Bum Contra 93 ± 12.48) but a slight difference in the ipsilesional side (Sham Ipsi 99.57 ± 13.01 versus CCI Bum Ipsi 63.20 ± 9.04) (Fig. 4C and D).

Using flow cytometry, we found that microglia were more likely to express the pro-inflammatory markers CD45, MHCII and CD172a (Veh MHCII^+^ 13.55 ± 4.91% versus Bum MHCII^+^ 21.59 ± 6.03%; Fig. 4E and F) after 3 days of bumetanide treatment. To confirm this, we measured interleukins in brain tissue and found a significant increase in IL-6 in the hippocampus of CCI animals treated with bumetanide (CCI Bum 21.94 ±6.03 pg/ml versus Sham 11.49 ± 0.91 pg/ml and CCI Veh 12.85 ± 3.89 pg/ml; Fig. 4G). Since TBI is characterized by inflammatory cell infiltration promoted by opening of the blood–brain barrier, we also looked at blood cells. The CD45^high^-CD11^low^ cells (e.g. possibly lymphocytes) were few in our samples and included not only neutrophils but also other innate immune cells (e.g. monocytes, macrophages and DCs) as reported in the second gating plot in Fig. 4E. However, systemic inflammation is not influenced by intraperitoneal bumetanide treatment, while intracortical inflammation is reduced by the same treatment.^36^ Thus, it is plausible that the effects of bumetanide may result from resident microglial cells and not infiltrating cells.

**Figure 5:**
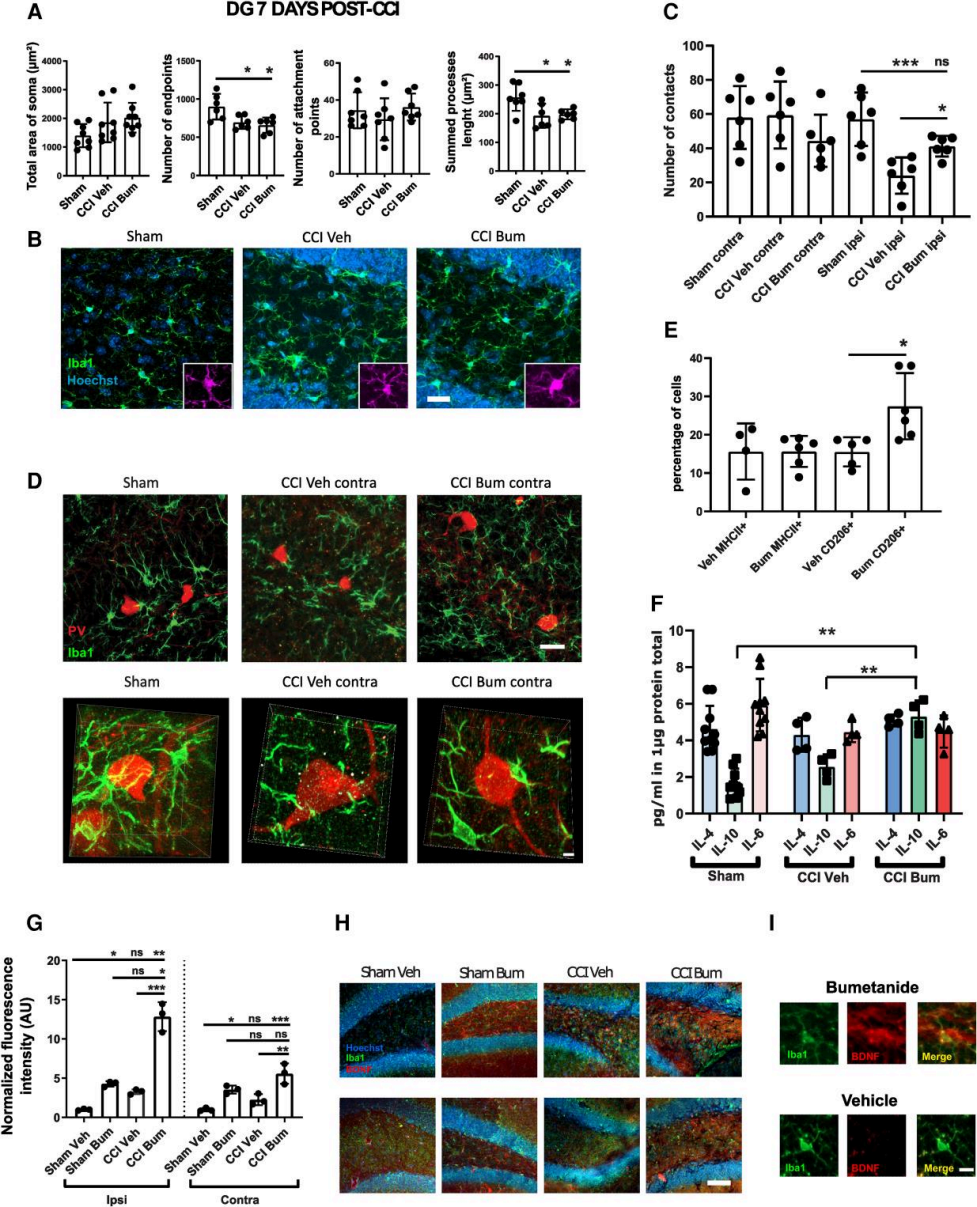
**Effect of bumetanide on microglia phenotypes and morphological changes after CCI in the DG at 7 days post-CCI**. (**A**) Total area of Iba1 + cells soma, quantifications of Iba1 + process end points, attachment points and length in the contralesional DG 7 days post-CCI. *n* = 5 animals, two slices per animal. (**B**) Iba1 immunostaining from sham-, CCI vehicle- and CCI bumetanide-treated animals in the contralesional DG. (**C**) Quantification of the number of contacts between microglia (Iba1 staining) and PV interneurons at 7 days post-CCI. *n* = 5 animals per condition, three slices per animal. (**D**) Example of PV and Iba1 immunostaining, from sham-, CCI vehicle- and CCI bumetanide-treated animals in the contralesional DG, 3D representations (*top*) and stack (*bottom*). (**E**) Analysis of microglia phenotypes by detection of MHCII^+^ (pro-inflammatory) and CD206^+^ (pro-phagocytosis) markers by flow cytometry on the contralesional side at 7 days post-CCI. *n* = 5–6 animals per condition. (**F**) Quantity of IL-4, IL-10 and IL-6 produced in the contralesional hippocampus. (**G**) Quantification of BDNF signal intensity within microglia of the ipsi- and contralesional brain of sham vehicle-, sham bumetanide-, CCI vehicle- and CCI bumetanide-treated animals. (**H**) Example of Iba1 and BDNF staining in DG of sham and CCI animals treated with bumetanide or vehicle. (**I**) Example of Iba1 and BDNF staining in a single microglial cell. *n* = 3, three slices per animal. Phenotype changes were analysed by Student's *t*-test. Number of contacts and morphological analysis (quantified using the ImageJ plugins Neurphology and SynapCountJ) were analysed using a one-way ANOVA with Dunnett's *post hoc* test. Interleukin production was analysed using a Kruskall–Wallis test for IL-10. *P < 0.05; **P < 0.01; ***P < 0.00; ns = not significant.

At 7 days post-CCI, we found less prominent morphological changes in microglia cells with no effect of bumetanide in both the ipsi- and contralateral side: a decrease in the number of end points (Sham 900.7 ± 167.9 versus CCI Veh 696.5 ± 86.72 versus CCI Bum 659 ± 100.4) and of process length (Sham 256.7 μm ± 46.75 versus CCI Veh 197.8 μm ± 17.17 versus CCI Bum 192.7 μm ± 42.21) (Fig. 5A and B). Despite this, the number of contacts between microglial cells and PV interneurons still differed for the ipsilesional side (CCI Veh 25.20 ± 12.30 versus CCI Bum 39.56 ± 5.85), without difference between sham and CCI bum (Sham Ipsi 57 ± 6.382 versus CCI Bum Ipsi 41.23 ± 2.472) (Fig. 5C and D). Surprisingly, bumetanide promoted the expression of the pro-phagocytosis marker CD206 by microglia (Veh CD206^+^ 16.79 ± 2.957% versus Bum CD206^+^ 27.44 ± 8.635%; Fig. 5E and F). In line with these results, we also found a significant increase of interleukin 10 (IL-10) expression in CCI animals treated with bumetanide compared to control and vehicle-treated animals (CCI Bum 5.32 ± 0.86 pg/ml versus Sham 1.69 ± 0.78 pg/ml and CCI Veh 2.57 ± 0.65 pg/ml; Fig. 5G).

These results suggest that bumetanide protects PV interneurons by increasing microglia contacts during the first post-traumatic week as well as by regulation of microglia phenotypes. This allows us to postulate that bumetanide treatment protects PV interneurons through anti-inflammatory and pro-survival mechanisms mediated by microglia.

### Bumetanide increases microglial BDNF expression

Microglia are a central source of BDNF, an important neurotrophin controlling neuronal survival and plasticity.^37–39^ Therefore, we measured the level of BDNF expression in microglial-like BV2 cells after 24 and 72 h of treatment. Twenty-four hours of bumetanide treatment led to a significant increase of BDNF levels (Ctrl 0.18 ± 0.02 pg/ml versus Bum 2.79 ± 0.18 pg/ml; Supplementary Fig. 3H). This effect was attenuated but still present after 72 h of treatment (Ctrl 0.19 ± 0.03 pg/ml versus Bum 0.31 ± 0.14 pg/ml; Supplementary Fig. 3I). In addition, we quantified the immuno-like intensity of BDNF in sham vehicle-, sham bumetanide-, CCI vehicle- and CCI bumetanide-treated animals and found a consistent increase in BDNF expression within Iba1-positive microglia in bumetanide-treated animals either on the contralesional (normalized value on sham vehicle: Sham Veh 1.00 ± 0.12 versus Sham Bum 3.55 ± 0.30 versus CCI Veh 2.27 ± 0.41 versus CCI Bum 5.60 ± 0.75) or the ipsilesional side (normalized value on sham vehicle: Sham Veh 1.00 ± 0.06 versus Sham Bum 4.28 ± 0.20 versus CCI Veh 3.28 ± 0.18 versus CCI Bum 12.86 ± 1.06; Fig. 5G–I). These results indicate that bumetanide induces BDNF expression, which may play an important role in the survival of PV interneurons.

### Bumetanide regulates post-CCI microglia process motility

**Figure 6:**
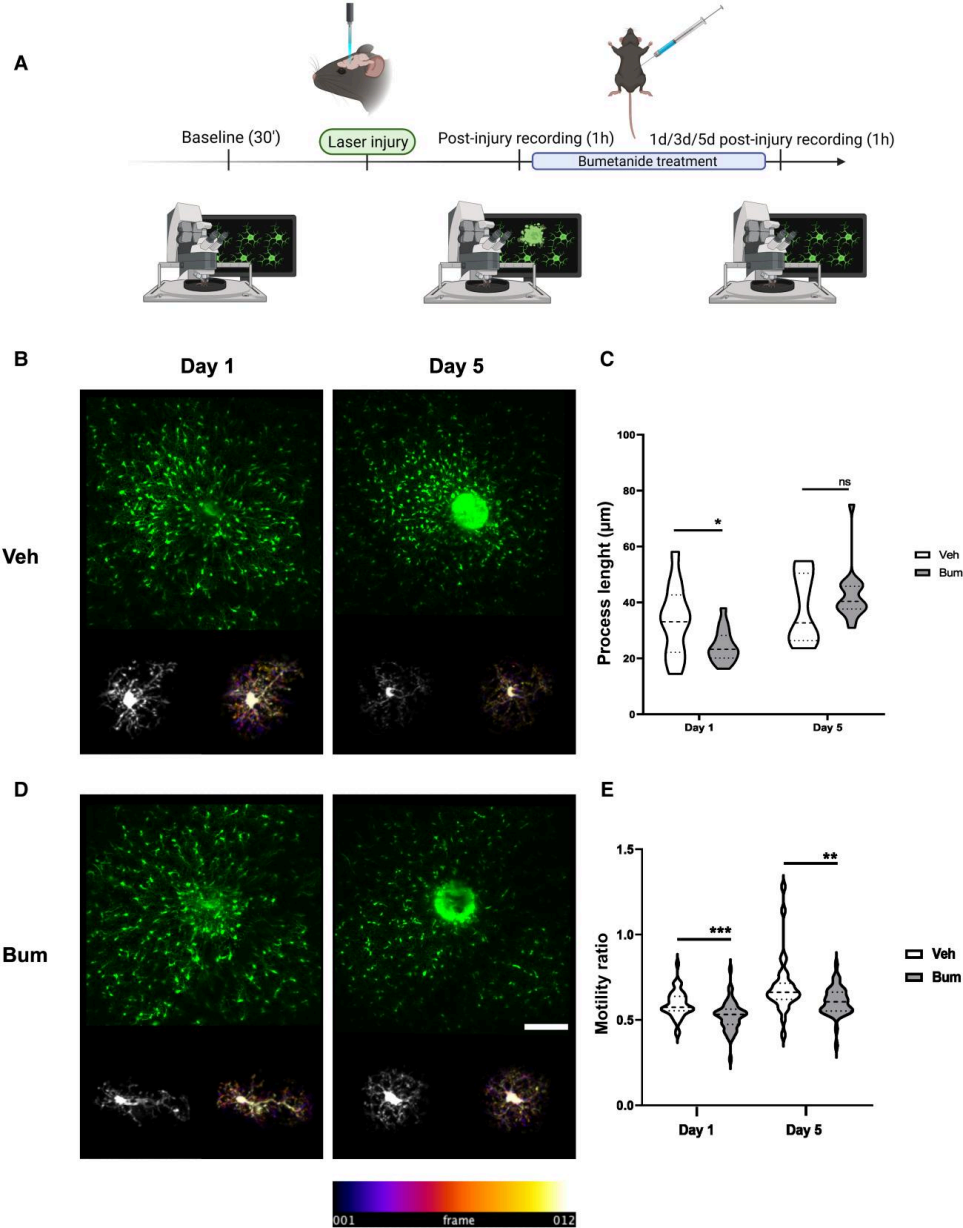
**Effect of bumetanide on post-injury microglia surveillance monitored by two-photon *in vivo* imaging**. (**A**) Schematic representation of the experimental timeline. (**B**) Maximal projection of image at 1 and 5 days after laser-induced focal injury. *Bottom*: A single microglia cell and the corresponding temporal colour-coded projection (recording time of 1 h at 5 min intervals). (**C**) Quantification of process length at 1 and 5 days after laser-induced focal injury in the presence and absence of bumetanide. (**D**) As for **B** in bumetanide (Bum)-treated animals. *Bottom*: A single microglia and the corresponding temporal colour-coded projection (recording time of 1 h at 5 min intervals). (**E**) Quantification of motility ratio at 1 and 5 days in the presence and absences of bumetanide. All data were analysed using unpaired Student's *t*-test. *P < 0.05.

To further investigate microglia *in vivo*, we performed longitudinal two-photon imaging in Cx3CR1^+/GFP^ mice following laser-induced injury on the posterior parietal cortex (see the Supplementary material for a full description of the procedure). Quantification of microglial process length and motility during the first week post-injury revealed that bumetanide induced a significant decrease in process length 1 day post-laser injury. This difference was not significant 5 days post-CCI (Veh Day 1 33.58 ± 3.66 versus Bum Day 1 24.43 ± 1.37; Veh Day 5 ± 3.69 versus Bum Day 5 42.51 ± 2.23;Fig. 6B–D). Interestingly, bumetanide induced a significant decrease in the motility ratio at both 1- and 5-days post-CCI (Veh Day 1 0.59 ± 0.1 versus Bum Day 1 0.52 ± 0.01; Veh Day 5 0.70 ± 0.03 versus Bum Day 5 0.61 ± 0.12; Fig. 6B, D and E). These experiments are in line with the effect of bumetanide on process length in CCI animals, and a decreased motility ratio is consistent with the increased number of contacts between microglia and PV interneurons.

### The effect of bumetanide is physiological state dependent

**Figure 7:**
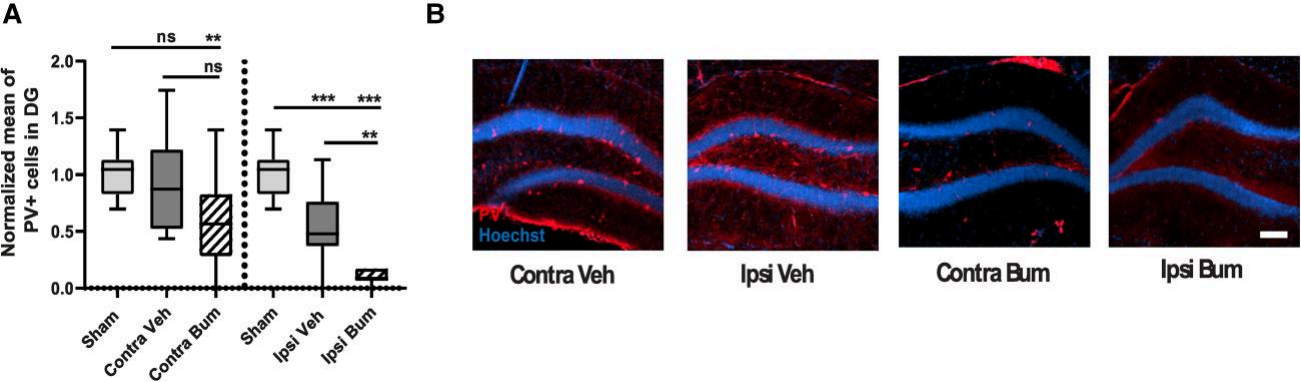
**Effect of pre-CCI bumetanide treatment on PV interneuron survival**. (**A**) Quantification of PV-positive cells 1 day post-CCI in the contralesional and ipsilesional DG of sham, CCI vehicle and CCI bumetanide animals treated for 7 days before CCI. *n* = 3, 5 and 5, respectively, three slices per animal. (**B**) PV labelling at 1 day post-CCI in the contralesional and ipsilesional DG of sham, CCI vehicle and CCI bumetanide animals, treated for 7 days before CCI (Scale bar = 100 μm). All datasets were analysed using one-way ANOVA with Dunnett's *post hoc* test. *P < 0.05; **P < 0.01; ***P < 0.00; ns = not significant.

Brain injury is paradoxically aggravated in microglia NKCC1-deficient mice.^36^ As under these conditions chloride uptake is impaired prior to the trauma, we asked if bumetanide treatment prior to CCI would have a different effect than when administered after CCI. Interestingly, administration of bumetanide during the week before CCI worsened CCI outcomes and, more particularly, PV survival. Indeed, bumetanide-treated animals showed a significant decrease in PV interneurons compared to sham- and vehicle-treated animals on the ipsilesional side (normalized value on sham: Sham 1 ± 0.05 versus CCI Veh 0.54 ± 0.09 versus CCI Bum 0.10 ± 0.02) and compared to sham on the contralesional side (normalized value on sham: Sham 1 ± 0.05 versus CCI Veh 0.90 ± 0.12 versus CCI Bum 0.57 ± 0.12) (Fig. 7A and B). These results indicate that the impact of bumetanide on PV-interneuron survival is dependent of the physiological state and the mechanism disclosed in this study is mainly relevant under pathological conditions.

## Discussion

How cognitive deficits observed in experimental models and clinical TBI develop following an initial concussion is not well understood.^5^ In this study, we were interested in how chloride homeostasis might be involved in post-traumatic changes in working memory.^40^ Theta rhythm is a potential electrophysiological biomarker of altered neuronal activity^41,42^ and is believed to synchronize activity both within local networks and between distal cortical regions involved in cognitive processing.^43,44^ In both humans and rats, the power of theta rhythm increases during the acquisition phase of spatial learning and object recognition. Treatment- or injury-induced inhibition of theta oscillations correlates with cognitive dysfunction.^29,45,46^ In agreement, we found a positive correlation between decreased theta-band power and poor performance in the spatial cognitive test after 1-month post-CCI. Interestingly, we found that an early transient treatment with the chloride cellular uptake inhibitor bumetanide was effective to counteract the long-term changes in both behaviour and theta oscillations.

Several studies link cognitive processes with normal adult neurogenesis in the hippocampus. This corresponds with the generation of functional neurons from adult neural stem cells. TBI can induce significant changes in the proliferation and maturation of granular cells.^47,48^ In the present study we found a significant increase in the number of Nestin-positive cells in both ipsi- and contralesional DG of mice 7 days post-CCI. Nestin is an RGL cell marker and represents a pool of quiescent cells that divide only occasionally.^16^ The division of these RGLs leads to the generation of intermediate progenitor cells (IPCs) that will undergo a limited number of rapid divisions before entering the neuronal differentiation pathway.^49^ IPCs are divided into two subtypes: (i) type 2a cells that are positive for Nestin and negative for DCX, an immature neuronal marker; and (ii) type 2b cells, positive for Nestin and DCX.^50^ IPCs then give rise to neuroblasts (type 3 cells), which express DCX.^51^ The increased number of Nestin-positive cells after TBI might intuitively suggest that there will be an increase in the number of newborn neurons. This is, however, not the case since we found a significant decrease in DCX-positive cells in both the contra- and ipsilesional side of the DG. These results indicate that TBI leads to an increase in the quiescent cell pool and a resulting decrease in the number of immature granule cells consistent with previous findings.^6^ Furthermore, treatment with bumetanide during the first week post-TBI significantly reduced the CCI-induced decrease in Nestin-positive cells, as well as increased the number of DCX-positive cells. These results suggest that the effects of bumetanide on cognitive performance could be related to positive effects on post-traumatic granule cell production. In addition, they may suggest a common upstream mechanism for the action of bumetanide.

GABA, especially released by PV interneurons,^7,52^ appears to play a central role in regulating the activity of RGLs by inhibiting their proliferation. Thus, post-traumatic changes in granule cells could be related to abnormal interneuron function. Indeed, we found loss of PV interneurons in the granular layer of the hippocampus, as early as 3 days post-CCI in both the ipsi- and contralesional side that was sustained at 7 days post-CCI and 1 month after trauma.^6^ This loss of PV interneurons may also contribute to abnormal brain activity in TBI.^53^

The increase of Nestin-positive cells and decrease in DCX cells that we detected after TBI can thus be related to the loss of PV interneurons, which results in less ambient GABA.^54,55^ In accordance with this assumption, treatment with bumetanide significantly counteracted the loss of PV interneurons and normalized the CCI induced change in Nestin and DCX-positive cells. As these cell populations were not significantly affected in sham animals by bumetanide a direct effect on progenitors may have less prominent importance.

Neuroinflammation is observed in both the acute and chronic stages of TBI.^12^ It appears to be responsible for both detrimental and beneficial effects by contributing to primary injury and secondary damage, but also facilitating tissue repair.^56^ Both astrocytes and microglia are important players in the mechanisms of brain inflammation and, in addition, display one of the highest expression levels of Nkcc1 in the brain. Thus, it is plausible that the post-traumatic effects produced of bumetanide could be related to an influence on the dynamics of inflammatory processes.^56,57^

Following this idea, we first studied the role of Nkcc1-mediated chloride uptake in astrocytes using conditional transgenic animals. Despite bumetanide having substantial effects on the morphology of GFAP-positive cells in both the contra- and ipsilateral sides in sham animals, the ablation of Nkcc1 in these cells did not rescue the loss of PV-positive interneurons. This indicates that astrocytes are not significantly involved in the bumetanide-induced increase in interneuron survival. Nevertheless, in this study we only monitored the first week post-CCI and we cannot exclude effects at more delayed stages of injury.

To define if CCI induced neuroinflammation through microglia could influence PV interneuron survival we first treated animals with minocycline, a second-generation tetracycline derivative that has an anti-inflammatory and neuroprotective action. Minocycline is known to have anti-inflammatory effect via the inhibition of microglial cells activation^58^ and induce microglial BDNF expression.^59^ Minocycline increased the survival of interneurons and the number of newborn neurons, suggesting that microglia inflammation is involved.

Interestingly, bumetanide treatment induced phenotypic changes in microglia and lead to a faster activation of microglial cells into a pro-inflammatory phenotype at early stages, which is required for cell debris removal and phagocytosis of apoptotic cells. At 7 days post-CCI, microglial cells of bumetanide-treated animals acquired pro-phagocytotic characteristics. Microglia in control animals acquired pro-inflammatory characteristics slightly later, but importantly it persisted more than 7 days post-CCI, resulting in a deleterious inflammatory state. Intriguingly, bumetanide increased the concentration of interleukin 6 (IL-6) 3 days post-CCI in line with a recent study using the same trauma model showing that hippocampal granule cells derived release of IL-6 is triggered by renewed microglial cells. This increase in neuronal survival allows adult neurogenesis to proceed normally and prevents behavioural deficits.^60^ One-week post-CCI, we detected higher levels of IL-10 in bumetanide-treated animals, along with a phenotype switch. Expression of IL-10 can promote neuronal and glial cell survival and dampen inflammatory responses via a number of signalling pathways.^61^ To further investigate if chloride uptake is important for microglia function, we knocked down the expression of Nkcc1 in primary microglia. We observed identical morphological changes and the same higher phagocytosis activity. This is interesting as microglia mediated phagocytosis is important for recovery as well as clearance of degenerated cells.^62^ Inhibition of phagocytosis can lead to increase neuronal damage and decrease neuronal cell viability.^63^

In addition, we found that bumetanide treatment leads to more contact sites between microglia and PV interneurons *in vivo*. This is consistent with previous results showing pro-survival signalling mediated by microglia contacts on interneurons.^15^ These results imply that the mechanism by which bumetanide rescues PV interneurons can be related to additional effects on microglia morphology. In accordance, bumetanide treatment leads to a significant increase of microglia processes and ramifications 3 days post-CCI. Moreover, it also significantly changes microglia process motility *in vivo*.

The neurotrophic factor BDNF is crucial for post-traumatic neuronal survival and plays a central role in microglia sensitive learning paradigms^64,65^ as well as microglia modulation of post-traumatic network function.^39^ Interestingly, we found here a significant increase of BDNF in microglia induced by bumetanide following CCI. There is a complex interplay between neurotrophic signalling and chloride homeostasis in neurons, e.g BDNF can normalize post-traumatic GABA_A_ mediated responses from depolarizing and hyperexcitable to hyperpolarizing but have the opposite effect under physiological conditions. This could contribute to neuronal survival under pathological conditions.^66,67^ Thus, changes in GABAergic transmission in combination with increased BDNF release from microglia and increased contact between microglia and interneurons could contribute to bumetanide induced increased survival of PV interneurons.

The effects observed here suggest that bumetanide directly acts on microglia. Indeed, recent work by Tόth *et al*.^36^ using microglia Nkcc1 deficient mice is in agreement with our results. In addition, they showed that systemic application of bumetanide had positive post-traumatic effects. In contrast, deletion of Nkcc1 in microglia resulted in more severe tissue damage after ischaemic stroke. Although these effects are compelling and indicate complicated actions of bumetanide, they do not provide a mechanistic view on the post-traumatic benefits of bumetanide. Moreover, Tόth *et al*.^36^ disrupted chloride uptake before induction of ischaemia. We obtained similar results, when we administered bumetanide already before induction of TBI. Thus, these results show a complex and context dependent effect of bumetanide and indicate that the mechanism disclosed in this study may apply to post-traumatic conditions.

Some limitations of this study could be addressed in future research. For example, we only used males^68–70^ and, as previously described,^71,72^ there are likely sex differences in clinical outcomes after TBI. On the other hand, no significant differences between males and females in lesion volume, neurodegeneration, blood–brain barrier alteration and microglia activation have been found in animal models.^73,74^ A second potential limitation is that we did not fully study the infiltrating immune cells due to the limited sample size. However, it would be interesting to address this, either as in Tóth *et al*.^36^ by using specific markers, e.g. CD3, CD20 and Ly-6G, or by using transgenic mice like CX3CR1creERT2×iDTR as in Willis *et al*.^60^

In conclusion, this study shows that a treatment targeting inhibition of the chloride co-transporter Nkcc1 can regulate the activation kinetics of microglia and provides a mechanistic link for the positive effect of bumetanide on post-traumatic cognitive decline. These results also open an additional avenue to better understand chloride uptake dependent pathophysiological mechanisms in the post-traumatic brain as well as to identify the elements involved in the development of long-term sequelae following TBI.

## Supplementary Material

awad132_Supplementary_Data

## Data Availability

The data that support the findings of this study are available from the corresponding author, upon reasonable request.
