# Supplementary material for: Bumetanide induces post-traumatic microglia–interneuron contact to promote neurogenesis and recovery
Source: Brain. 2023 Apr 21;146(10):4247–61. doi: 10.1093/brain/awad132 (PMC10545516; doi:10.1093/brain/awad132)
Supplement: awad132_Supplementary_Data [file awad132_supplementary_data.pdf]

Supplementary Material and Methods

Experimental Animals

The French ethical committee approved all experimental procedures (N°: APAFIS#2797). Wild-type mice had a C57bl6-J background. Three transgenic lines were used: Nestin-GFP[1], hGAFP-Cre x NKCC1 flox and B6.129P-Cx3cr1tm1Litt/j[2] and were all maintained on a mixed genetic background. All animal experiments also complied with the ARRIVE guidelines and were carried out in accordance with the U.K. Animals (Scientific Procedures) Act, 1986 and associated guidelines, EU Directive 2010/63/EU for animal experiments. The 3Rs principle was applied: i) Replacement – no substitute in vitro model can study inflammation in different region of the brain simultaneously following brain injury; ii) Reduction – the same animals were used to performed western blot analysis and the ELISA assay to reduce the number of animals; iii) Refinement – signs of animal suffering and welfare were monitored. Indeed, animals were excluded when clinical signs of pain and discomfort were observed, including prostrated animals with absence of movement in the cage and/or hunched posture, ungroomed appearance, weight loss higher than 20%, dehydration, decreased urine/fecal output, piloerection, chronic porphyrin staining around eyes, nose or forelimbs and rapid respirations. Only males were used, and they were housed individually in an enriched environment, maintained in a 12 h light/12 h dark cycle with a controlled temperature (23 ± 2°C), food and water being given ad libitum. All experiments were performed in single-blind.

In vivo electrophysiological recordings

Scalp electroencephalographic (EEG) recordings were performed in freely moving mice 3 weeks post-CCI. Telemetric recording electrodes were implanted at the level of the posterior parietal cortex at 2 mm posterior to and 1.5 mm left of the bregma. A reference electrode was placed rostral in the cerebellum. After a 72-hour recovery, EEG (amplified 31,000, filtered at 0.1–120Hz pass, acquired at 1000Hz) was monitored using a telemetric system (EMKA TECHNOLOGIES S.A.S) for 3 days, 24 hours per day.

For in vivo intrahippocampal multisite recordings one-month-old male mice (C57bl6-J, n=7) were anesthetized using isoflurane (4% for induction and stabilized at 2/2,5%) mixed with air and enriched with oxygen (0.3 %) then positioned in in a stereotaxic frame (David Kopf Instruments®). Body temperature was monitored throughout the procedure using a rectal probe

and maintained at 37 ± 2°C with a heating pad (Harvard Apparatus®). Animals received subcutaneous injections of Buprenorphine (0,03 mg/kg) and carprofen (5mg/ml). A metallic head-plate (Neurotar®), was attached to the skull using dental cement (Opti-Bond de Kerr) at the level of the posterior parietal cortex and the recording coordinates 2 mm posterior to and 1.5mm left of the bregma were marked using an indelible marker. Animals were monitored during the following week to make sure the recovery was successful. After a 72 hours recovery, animals were trained in the Mobile HomeCage® (MHC V5, Neurotar®) for the next 7 days. Two days prior to recording, animals were anesthetized (isoflurane 4% for induction and stabilized at 2/2,5%) and received subcutaneous injections of Buprenorphine (0,03 mg/kg) and carprofen (5mg/ml). Two craniotomies were performed using a 1mm diameter drill and the recording sites protected using a silicon cast (Kwik-Cast™). Ten days after surgery, trained animals were placed in the Mobile HomeCage® (MHC V5, Neurotar®) and a one shank 16 channels linear probe inserted 1 mm ventral (A1x16-3mm-100-177, Neuronexus). Animals were recorded 3 times 10 minutes. Recordings were acquired using the Allego software (SmartBox Pro™, Neuronexus) and a sampling rate of 30K Hz per channel.

Recordings were visually inspected using NeuroExplorer (version 5.305), and artifacts (e.g., disconnections, movement artifact) were manually rejected from all subsequent analyses. Raw data were then down sampled to 1,000 Hz and filtered for 50 Hz line noise using a Butterworth band stop IIR filter. Animal temperature as well as movement were used to determine active and non-active time windows. Delta, Theta and Gamma band were operationally defined as1-4 Hz, 4–12 Hz ;30-80 Hz[3], and spectral power estimates across frequency bands (2–80 Hz, 80 logarithmically spaced points) were calculated using Morlet wavelets [cycles = 6; (56)] and first normalized using the ratio of power in the first minute to the last minute within each recording session. The mean normalized power of active time windows was subtracted from non-active for each recording.

Individual units were isolated from multisite LFP recordings using Spyking-Circus (https://github.com/spyking-circus)[4]. Clusters with >1% of ISIs violating the refractory period (<2 ms) or appearing otherwise contaminated were manually removed from the dataset. Pairs of units with similar waveforms and coordinated refractory periods in the cross-correlogram were combined into single clusters. Unit position with respect to electrode sites was characterized as the average of all electrode site positions weighted by the wave amplitude on each electrode.

The Single-unit activity were further classified into wide-spiking (WS) and narrow-spiking (NS) units[5] based on the spike waveform, auto-correlogram, and cross-correlogram. Units with

short half-amplitude width, short trough-to-peak time, were classified as NS units; the rest of the units were classified into WS units (Fig S4). NS and WS presumably correspond to putative inhibitory and excitatory units, respectively[6,7].

Immunohistochemistry

Mice were transcardially perfused with cold phosphate buffer saline (PBS 1X) prior to a 3% paraformaldehyde solution (AntigenFix, Diapath®). Brains were post-fixed overnight in 3% paraformaldehyde at 4°C and cells were fixed in 3% paraformaldehyde at RT for 5 mins. Sections and cells were permeabilized and blocked in PBS with 0.3% Triton X-100 and 5% normal goat serum (NGS) for 1 h at room temperature. Incubation with primary antibodies diluted in PBS with 5% NGS and 0.3% Triton X-100 was carried out at 4°C overnight using rabbit anti-doublecortin (ab18723, Abcam, 1:1000), mouse anti-parvalbumin (p3088, Sigma, 1:500), rabbit anti-Iba1 (W1W019-19741, Wako, 1 :500), mouse anti-GFAP (MAB360, Merck Millipore, 1 :500), mouse anti-BDNF antibody (MAB#918), mouse anti-RFP antibody (MA5-15257, Thermofisher Scientific, 1:500) and chicken anti-GFP antibody (AB_2307313, Avès Labs, 1:300). Slices and cells were incubated with the corresponding Alexa Fluor-conjugated secondary antibodies diluted in PBS (Thermo Fisher Scientific, 1 :500) for 2 h at room temperature and finally counterstained for 1 min with Hoechst 33258 (10 µg/mL in PBS, Sigma-Aldrich). Images were taken using a confocal microscope LSM-800 Zeiss (28148). Morphology analysis was performed with the ImageJ® plugin Neurphology.

PV microglia contacts quantification

Confocal stacks with double immunofluorescent labeling (Parvalbumin and Iba1) were acquired using a LSM-800 Zeiss confocal microscope Plan Flour 40x/1,30 NA oil immersion objective. Optical sections with a step size of 0.3 mm to a total of 15 mm were collected from the ipsi- and contralesional hippocampi of mice treated and non-treated with bumetanide, at 3 and 7 dpCCI. Image stacks were 3D-deconvoluted using the Autoquant X software (Media Cybernetics Inc). Images were then reconstructed in 3D with the Imaris Software. 3D surfaces were rendered from each of the corresponding channels for microglia (green channel) and interneurons (red channel) and the contact points were defined as surfaces within 0.5 µm from each other. Then, contacts between microglia and PV interneurons were quantified with the ImageJ® plugin SynapCountJ from microglial processes of at least 0.5 long segments and visually inspected.

Live Imaging

5-month-old male mice (B6.129P-Cx3cr1tm1Litt/j, n = 5) were anesthetized (ketamine (90 mg/kg) and xylazine (4.5 mg/kg), IP). Animals received subcutaneous injections of dexamethasone sodium phosphate (2 µg/g) and carprofen (5 mg/ml). The bone fragment was removed using a 0.45 mm diameter drill. A coverslip was placed on top of the cranial window. A metallic head-plate was attached to the coverslip forming a frame for the window. Animals were monitored during the following weeks ensure a successful recovery.

1 month after surgery, animals were trained for 5 days before the imaging session on a Mobile HomeCage® (MHC V5, Neurotar®).

Trained awake animals were head fixed on a Mobile HomeCage® platform (MHC V5, Neurotar®) under a custom-built two-photon microscope (Femtonics®) and imaged longitudinally using a Nikon Apo LWD 25X objective with 1.10 numerical aperture for 5 days. The imaging protocol consisted of excitation at 920 nm using a Mai Tai® Ti:Sapphire Ultrafast Laser (Spectra-Physics) and emission collected at 530/30. An image stack with a 5 mm step size covering 300 mm was acquired. Each animal had a 30 min baseline recording. Injury was then achieved by focal excitation of 1 cell for 20 s at 75 µs/pixel at 100% laser intensity. After the injury, animals were IP injected with either the vehicle or bumetanide and z-stacks were collected covering the entire injury area (around 300 µm) with 5 min intervals for 1 h. Animals were IP injected twice per day with the vehicle or bumetanide and imaged using the protocol described daily from day 1 to day 5 post injury.

To compute microglia cell dynamics, we designed a semi-automated procedure using Python. First, the 4D image stacks (3D + time) were loaded into the Napari viewer for a manual selection of the cells of interest. Sub-volumes were then cropped around each cell before undergoing a series of spatial registrations using pystackreg (G. Lichtner, pystackreg, (2022), https://github.com/glichtner/pystackreg) ported from the ImageJ TurboReg plugin[8]. The aim of these registrations was to correct translational, rotational and shearing drift that could be observed during acquisition. After maximum projection along the z-axis, the user was prompted to define regions of interest (ROIs) around each microglia and signal outside of these ROIs was discarded. To segment the microglia, an intensity threshold was determined and applied according to Li's method[9] and the cell dynamics was evaluated by calculating the percentage of binary pixels changing in value from one time point to another[8].

Behavioral tests

1 month post-CCI, mice were tested on different tasks using the object recognition paradigm to test the individual components of episodic-like memory, namely the novel object recognition (NOR) and the object displacement task (ODT)[10]. Animals were habituated to the testing room 24 h before testing. For NOR, mice were placed in the center of a maze (Noldus apparatus®, 38.5 cm x 38.5 cm) and allowed to freely explore the space for 10 min. Then, two identical objects were added for a 3 min exploration time. Finally, after a 3 min retention time, one of the objects was replaced and the time of exploration was measured for a 3 min period. This test was used to assess short-term memory. The same experiment was also performed with a 1-hour retention time to test long-term memory.

For the ODT, mice were placed in the center of the same maze for 3 min, then 2 identical objects were added for 3 min. After a 3 min retention time, one of the objects was moved and the time of exploration of each object was recorded.

Mice were also tested for pure spatial memory learning using a Barnes maze (BM) test. Briefly, the Barnes maze is a black, metallic, circular table with a diameter of 100 cm elevated 60 cm from the ground. In the periphery there are 20 evenly spaced holes, where 19 of these holes are "false escapes" in which the mice cannot effectively hide. One hole contains the escape box (target hole), which leads to a dark recess below the table. 1-month post-CCI, mice were trained twice daily for a duration of 4 days. On trial probe day (day 5), the target box was replaced with a false escape. The distance travelled, the number of errors, the time spent in the target zone and the latency to reach the target hole were measured.
Finally, mice were tested for anxiety using the elevated plus maze (EPM) test. The maze from Ugo Basile company® is composed of 2 open arms and 2 closed arms that extended from a central platform (5 cm x 5 cm). Each arm is 50 cm long and are at a height of 50 cm above the floor, with the closed arms being surrounded by a 15 cm height wall. For each test, mice were placed in the center square facing an open arm and allowed to move freely for 5 mins. Entries and time spent in the open arms, closed arms and center were monitored. Recording and analyses was done using the Ethovision software (Noldus®).

Western blotting

Western blotting was performed as described in Goubert *et al.*[6]. Membranes were exposed overnight at 4°C to an NKCC1-specific antibody (DSHB Hybridoma Product T4) diluted

1:2000 in blocking solution. Chemiluminescent detection was performed using the ECL-plus kit (Pierce Biotech®). We measured signal intensities with the image analysis software G box (Syngene®). Then, membranes were stripped and probed with rabbit anti-α-tubulin (Sigma®, 1:10 000). Quantification was performed using the Gel Plot Analyzer plugin (Fiji®).

ELISA

Quantification of mature BDNF (mBDNF) was performed with the mBDNF Rapid ELISA Kit (Biosensis®, BEK-2211-1P/2P – sandwich ELISA - Thebarton, SA, Australia) following the manufacturer's protocol. The Quality Control sample ranged between 175-325 pg/ml and the mBDNF standard ranged from 7.8-500 pg/ml. Concentrations were determined using the FLUOstar® OPTIMA microplate reader (BMG Labtech, France). Microglia interleukines production was analyzed the same way with the Mouse Interleukin 6 ELISA Kit (Biosensis®, BEK-2043-1P) and the Mouse Interleukin 10 ELISA Kit (Biosensis®, BEK-2046-1P).

Cell culture

- Cell lines:

As described previously[20], BV2 cells were cultured in Gibco Dulbecco's Modified Eagle Medium (DMEM) media containing 10% fetal bovine serum, 100 IU/mL penicillin, and 100 mg/mL streptomycin for amplification. After seeding, we cultured BV2 cells in DMEM media containing only 1% fetal bovine serum, 100 IU/mL penicillin, and 100 mg/mL streptomycin since serum deprivation triggers cellular differentiation. Plates were incubated at 5% CO^2 and 37 °C. BLPS (50 ng/mL), bumetanide (40 nM) or LPS + Bumetanide were added to the culture media for either 24 or 72 h.

- Primary culture:

Microglia were isolated from cultures of newborn CX3CR1-CreERT2 mice brain and the tissue was dissociated by trituration with trypsin (Gibco, United States) as described previously[21]. In brief, isolated cerebral cortices from newborn CX3CR1-CreERT2 mice were taken off the meninges, minced in DMEM complete medium (Gibco, United States) with 10% fetal bovine serum (Gibco, United States) and 1% antibiotics before being dissociated by trituration in 0.25% trypsin/EDTA. Cells were plated in 75 cm^2 plastic culture flasks containing 15 ml complete medium with 10% fetal bovine serum at a density of 2×10^6 cells/ml. Culture flasks

were vigorously agitated on a rotary shaker twice for 10 minutes after 10 days of culture. Here, GFAP+ astroglia remain adherent to the flasks and the resulting cell suspension, rich in microglia, was placed in new plastic flasks (10^5 cells/ml) and allowed to adhere at 37°C. Cells were then plated into LAB-TEK II (Thermo Fisher Scientific) at a density of 2.5×10^4 cells/well for the phagocytosis assay and morphology analysis.

- Transfection:

pSico-shNKCC1[22] (Addgene, #83027) and a pCAG-RFP plasmid were co-transfected into microglia cultured from newborn CX3CR1-Cre ERT2 using Glial Mag (OZ Bioscience). 4-hydroxytamoxifen (Merck, Nº5082250001) was then added to the culture media at a final concentration of 2 µM for 3 days.

- Phagocytosis Assay

Microglia were seeded into Labtech plates at a density of 2.5×10^4 cells/well and cultured for 3 days under different conditions: co-transfected with pSico-shNKCC1 and pCAG-RFP and treated with bumetanide and tamoxifen, transfected with pCAG-RFP and treated with tamoxifen, or co-transfected with pSico-shNKCC1 and pCAG-RFP and treated with tamoxifen. Latex beads-Rabbit IgG-DyLight 633 Complex (Cayman chemical®, No.601490) were directly added to the pre-warmed culture medium (final dilution of 1:250). After incubating the DMEM medium containing latex beads with the treated microglia for 2 h, cells were fixed, stained with Hoechst and incubated in an anti-Iba1 antibody for subsequent fluorescence microscopy.

Supplementary Results

Deletion of Nkcc1 in Gfap-positive astrocytes does not show similar effect than bumetanide.

Although CCI induced robust changes at 3, 5 and 7 days in GFAP positive astrocytes we did not observe a significant effect of bumetanide on CCI induced changes in astrocyte morphology on either ipsi and contralateral side at 3 and 5 days after treatment (Fig S1A, B, C and D). We only did find however significant effects of bumetanide on the contralesional DG at 7 days post-CCI where bumetanide decreased the length of astrocyte processes (CCI Veh 290 µm ± 10.91 vs CCI Bum 147 µm ± 22.17, Fig S1F). The treatment also decreased the soma's area (Sham 1951 µm² ± 633.6 vs CCI Veh 3232 µm² ± 805 vs CCI Bum 1888 µm² ± 465.8) and the number of endpoints (Sham 895.5 ± 108,2 vs CCI Veh 594.4 ± 130.4 vs CCI Bum 957.2 ± 155.9; Fig S1G and H).

To investigate whether the effect of bumetanide on adult neurogenesis depends on Nkcc1 in astrocytes, we monitored the effect of CCI in transgenic mice where Nkcc1 was specifically deleted in astrocytes (GFAP-Nkcc1 KO), thus mimicking the effect of bumetanide on astrocytes (Fig S2 A and B). Interestingly, there was no impact on adult neurogenesis in hGFAP-Nkcc1 KO mice, neither in the contralesional DG (Sham +/+ 100 % ± 16.03 vs Sham Cre/+ 100 % ± 8.91 and Contra +/+ 80.1 % ± 10.6 vs Contra Cre/+ 80.7 % ± 19.90) nor in the ipsilesional DG (Ipsi +/+ 47.1 % ± 16.85 vs Ipsi Cre/+ 44.9 % ± 12.46, Fig S2C). Similarly, we found no effect of Nkcc1 deletion in astrocytes in CCI-induced PV interneuron decrease in both the contra- (Sham +/+ 100 % ± 15,96 vs Sham Cre/+ 100,00 % ± 28,43 and Contra +/+ 82,34 % ± 22,20 vs Contra Cre/+ 79,34 % ± 24,22) and ipsilesional DG (Ipsi +/+ 23,94 % ± 20,81 vs Ipsi Cre/+ 25,44 % ± 21,40), (Fig S2D).

We also compared the effect of bumetanide treatment and Nkcc1 deletion in astrocytes in hGFAP-Nkcc1 knock-out (KO) mice 7 dpCCI. While bumetanide had no effect on the number of processes in WT sham animals (Sham +/+ 7.90 ± 0.77 vs Sham +/+ Bum 5.55 ± 1.51), it caused a significant increase in both the ipsi- and contralesional DG (Ipsi +/+ 9.76 ± 1.21 vs Ipsi +/+ Bum 14.10 ± 1.98 and Contra +/+ 7.37 ± 1.16 vs Contra +/+ Bum 16.76 ± 4.02). hGFAP-Nkcc1 KO animals showed an increase in the number of processes in sham animals (Sham +/+ 7.90 ± 0.77 vs Sham Cre/+ 10.64 ± 0.73) but no effect on either the ipsi- or contralesional side after Bumetanide treatment (Ipsi +/+ 9,76 ± 1,21 vs Ipsi Cre/+ 8,45 ± 1,22 and Contra +/+ 7.37 ± 1.16 vs Contra Cre/+ 7.52 ±0.68) (Fig S2F). In addition, bumetanide

treatment induced a decrease of astrocyte soma size in WT controls (Sham +/+ 438.2 µm² ± 121.4 vs Sham +/+ Bum 193.1 µm² ± 67.36) and a significant increase on both the ipsi- and contralesional sides post-CCI (Ipsi +/+ 495.4 µm² ± 189.7 vs Ipsi +/+ Bum 885.7 µm² ± 112.5 and Contra +/+ 331.6 µm² ± 103.6 vs Contra +/+ Bum 655.9 µm² ± 114.9). Conversely, in hGFAP-Nkcc11 KO we observed a significant increase of astrocyte soma size in sham animals (Sham +/+ 438.2 µm² ± 121.4 vs Sham Cre/+ 713.3 µm² ± 73.82) but no change on either the ipsi- or contralesional sides post-CCI (Ipsi +/+ 495.4 µm² ± 189.7 vs Ipsi Cre/+ 514.0 µm² ± 73.69 and Contra +/+ 331.6 µm² ± 103.6 vs Contra Cre/+ 395.2 µm² ± 73.48) (Fig S2G).

There is a complex interplay between astrocyte and microglia activation during pathophysiological conditions. For this reason, we also investigated the effect of GFAP-Nkcc1 KO on microglia. After 7 days of bumetanide treatment, the number of microglial processes in WT animals did not differ between sham or contralesional DG of CCI animals (Sham +/+ 55.53 µm ± 12.56 vs Sham +/+ Bum 49.11 µm ± 24,52 and Contra +/+ 38.82 µm ± 6.241 vs Contra +/+ Bum 34.01µm ± 9.768) (Fig. S2H). However, it led to an increase in the number of microglial processes on the ipsilesional side (Ipsi +/+ 36.50 ± 6.025 vs Ipsi +/+ Bum 55.57 ± 12.40). In hGFAP-Nkcc1 KO mice, there was no change in the number of processes in either the sham condition (Sham +/+ 55.53 ± 12.56 vs Sham Cre/+ 50.92 ± 16.25) or on the ipsilesional side (Ipsi +/+ 36.50 ± 6.025 vs Ipsi Cre/+ 41.65 ± 4.984) of CCI animals. However, a significant increase was observed on the contralesional side of CCI animals (Contra +/+ 38.82 ± 6.241 vs Contra Cre/+ 52.78 ± 10.42). The average size of microglial soma was significantly reduced after 7 days of bumetanide treatment in WT sham mice (Sham +/+ 318,1 µm² ± 70.85 vs Sham +/+ Bum 245.5 µm² ± 47.64) on the ipsilesional side (Ipsi +/+ 750.3 µm² ± 227.0 vs Ipsi +/+ Bum 419,6 µm² ± 54.48), but there were no changes on the contralesional side (Contra +/+ 367.7 µm² ± 65.99 vs Contra +/+ Bum 379.0 µm² ± 23.37). In hGFAP-Nkcc1 KO animals there were no changes in either the sham condition (Sham +/+ 318.1 µm² ± 70.85 vs Sham Cre/+ 329.2 µm² ± 53.61), on the ipsilesional side (Ipsi +/+ 750.3 µm² ± 227.0 vs Ipsi Cre/+ 597.0 µm² ± 145.7), or the contralesional side (Contra +/+ 367.7 µm² ± 65.99 vs Contra Cre/+ 353.8 µm² ± 68.58) (Fig S2H).

These results indicate that the depletion of Nkcc1 in astrocytes produced significantly different effects on astrocyte morphology than bumetanide treatment. In addition, the positive effects that occur in neurogenesis and interneuron survival after bumetanide treatment might not be mediated by astrocytes.

Bumetanide induces Nkcc1 expression and modify the morphology of a microglial cell line.

To investigate how bumetanide can influence microglia morphology and their trophic actions, we used a BV2 murine microglial cell line. 24 hours of treatment with bumetanide did not show significant changes in Nkcc1 expression (normalized value on control: Ctrl 1 ± 0.17 vs Bum 0.82 ± 0.29, n= 12 wells per conditions, Fig. S3A). However, after 72 hours we detected a significant increase (Ctrl 1 ± 0.18 vs Bum 2.24 ± 0.99, Fig S3A). We then assessed the morphology of BV2 cells. We observed that 24h of bumetanide treatment induced a significant increase in average cell size (Bum 3161 µm² ± 371.3 vs Ctrl 1993 µm² ± 164.1, Fig S3B and D). At 72h this effect was not significant (Bum 3100 ± 414.1 vs Ctrl 1974 ± 231.5, Fig S2E and G). The effect of bumetanide on the number of processes was the opposite. While no effect was found at 24 h (Bum 15.90 ± 4.73 vs Ctrl 22.08 ± 6.96 Fig S3B and C), bumetanide induced a significant decrease after 72 h (Bum 14.59 ± 5.205 vs Ctrl 35.16 ± 10.84, Fig S3F and G). These results further indicate that bumetanide can regulate microglial morphology by modulating chloride co-transporter Nkcc1 expression.

Legends to Supplementary Figures and Table

FIG S1 | Astrocyte morphological changes after CCI in the contralesional side 7 dpCCI and effect of bumetanide on major inflammatory cells.

(A) Impact of CCI and bumetanide on GFAP staining intensity and cell number in 3, 5 and 7 dpCCI ipsi and contralesional DG. (B) Total area of GFAP+ cell soma, quantifications of GFAP process endpoints, attachment points and length in the contralesional DG, 7 dpCCI. **(C)** GFAP immunostaining from sham, CCI-vehicle and CCI-bumetanide treated animals in the contralesional DG 7 dpCCI. All sets of data were analyzed using Brown-Forsythe ANOVA test with Dunnett's post hoc test or using a t-test with Welch's correction. *p < 0.05; **p < 0.01; ***p < 0.001. Calibration bar is 25 μm.

FIG S2 | Astrocyte morphological changes after CCI in the contralesional side 7 dpCCI and effect of bumetanide on major inflammatory cells.

(A) Western blot and **(B) quantification of** Nkcc1 and Gfap protein expression normalized to the ubiquitous marker GAPDH in WT (fl/fl +/+) and GFAP-Nkcc1 KO mice (fl/fl Cre/+) hippocampus. **(C)** Quantification of DCX-positive cells 7 dpCCI in the DG of sham Nkcc1 fl/fl Gfap +/+, sham Nkcc1 fl/fl Gfap Cre/+, CCI Nkcc1 fl/fl Gfap +/+ and CCI Nkcc1 fl/fl Gfap Cre/+. **(D)** Quantification of PV-positive cells 7 dpCCI in the DG of sham Nkcc1 fl/fl Gfap +/+, sham Nkcc1 fl/fl Gfap Cre/+, CCI Nkcc1 fl/fl Gfap +/+ and CCI Nkcc1 fl/fl Gfap Cre/+. **(E)** Quantification of the number of processes and average soma size of astrocytes (Gfap+ cells) in Nkcc1 fl/fl Gfap-Cre/+ and Nkcc1 fl/fl Gfap +/+ mice at 7 dpCCI. **(F)** Quantification of the number of processes and average soma size of microglia (Iba1+ cells) in Nkcc1 fl/fl Gfap-Cre/+ and Nkcc1 fl/fl Gfap +/+ mice at 7 dpCCI. n = 5 animals, 2-3 slices per animal. Morphological analyses were quantified using the ImageJ plugin Neurphology. All sets of data were analyzed using Brown-Forsythe ANOVA test with Dunnett's post hoc test or using a t-test with Welch's correction. *p < 0.05; **p < 0.01; ***p < 0.001.

FIG S3 | Microglia expresses Nkcc1, their morphology and Bdnf production are modified by bumetanide treatment in vitro.

(A) The left panel represents Nkcc1 protein expression normalized to the ubiquitous marker α-tubulin in BV2 cells 24 h after LPS, Bumetanide or LPS + Bumetanide treatment. On the right panel, normalized Nkcc1 expression is shown after 72 h of the same treatments. **(B)** Average size of BV2 somas in the control condition and after a 24 h bumetanide treatment. **(C)** Number of processes in BV2 cells in the control condition and after a 24 h bumetanide treatment. **(D)** Iba1 immunostaining of BV2 cells cultured and fixed on LAB-TEK plates in the control condition and after a 24 h bumetanide treatment. **(E)** Same as **(B)** after 72 h of treatment. **(F)** Same as **(C)** after 72 h of treatment. **(G)** Same as **(D)** after 72 h of treatment. **(H)** Measurement of BV2 Bdnf levels after 24 h and **(I)** 72 h of treatment. n = 12 wells per conditions. 7-8 randomly selected cells per well. The sets of data from A to F were analyzed using T-tests. Sets of data from H to I were analyzed using a Mann-Whitney test $*p < 0.05$; $**p < 0.01$; $***p < 0.001$.

FIG S4 | Representative pictures of isolated unis and EEG power spectra

(A) Representation templates for wide-spiking (left) and narrow spiking (right) units with corresponding autocorrelogram (below). (**B**) EEG of (bandpass 1-14 Hz) Power spectra of active, non-active and difference between these two states in a Sham animal. The subtracted power spectra clearly show a peak in the theta range. Autocorrelogram ± 50 ms, calibration bar for units 0.5 ms.

Supplementary Table 1 | Descriptive statistics of all data sets

Descriptive statistics of data sets per figure including the normality of the data sets, the test used, the groups compared, the difference between the group means, the SE of diff, the number of values per group, the DF, the statistically significant difference and the adjusted p-value.

SE of diff = Standard error of the difference between mean; DF = Degrees of freedom

References

1. Mignone JL, Kukekov V, Chiang AS, Steindler D, Enikolopov G. Neural stem and progenitor cells in nestin-GFP transgenic mice. *J Comp Neurol*. 2004;469(3):311-324. doi:10.1002/cne.10964

2. Yona S, Kim KW, Wolf Y, et al. Fate mapping reveals origins and dynamics of monocytes and tissue macrophages under homeostasis. *Immunity*. 2013;38(1):79-91. doi:10.1016/j.immuni.2012.12.001

3. Cohen MX. *Analyzing Neural Time Series Data: Theory and Practice*.; 2014. doi:10.7551/mitpress/9609.001.0001

4. Yger P, Spampinato GL, Esposito E, et al. A spike sorting toolbox for up to thousands of electrodes validated with ground truth recordings in vitro and in vivo. *eLife*. 7:e34518. doi:10.7554/eLife.34518

5. Watson BO, Levenstein D, Greene JP, Gelinas JN, Buzsáki G. Network homeostasis and state dynamics of neocortical sleep. *Neuron*. 2016;90(4):839-852. doi:10.1016/j.neuron.2016.03.036

6. Csicsvari J, Hirase H, Czurko A, Buzsáki G. Reliability and state dependence of pyramidal cell-interneuron synapses in the hippocampus: an ensemble approach in the behaving rat. *Neuron*. 1998;21(1):179-189. doi:10.1016/s0896-6273(00)80525-5

7. Sirota A, Montgomery S, Fujisawa S, Isomura Y, Zugaro M, Buzsáki G. Entrainment of neocortical neurons and gamma oscillations by the hippocampal theta rhythm. *Neuron*. 2008;60(4):683-697. doi:10.1016/j.neuron.2008.09.014

8. Thévenaz P, Ruttimann UE, Unser M. A pyramid approach to subpixel registration based on intensity. *IEEE Trans Image Process*. 1998;7(1):27-41. doi:10.1109/83.650848

9. Li CH, Lee CK. Minimum cross entropy thresholding. *Pattern Recognition*. 1993;26(4):617-625. doi:10.1016/0031-3203(93)90115-D

10. Inostroza M, Brotons-Mas JR, Laurent F, Cid E, de la Prida LM. Specific impairment of “what-where-when” episodic-like memory in experimental models of temporal lobe epilepsy. *J Neurosci*. 2013;33(45):17749-17762. doi:10.1523/JNEUROSCI.0957-13.2013

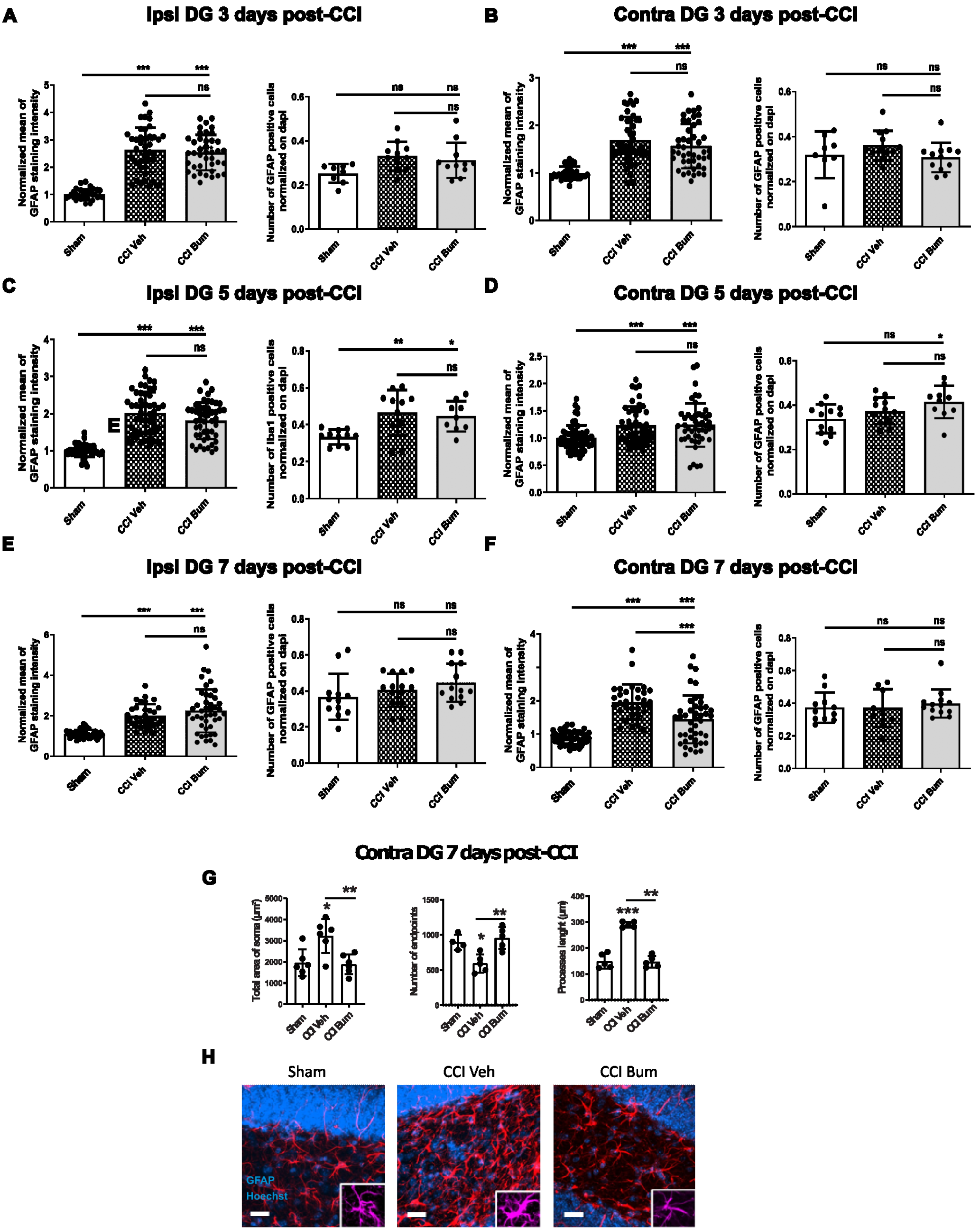

Supplementary Figure S1

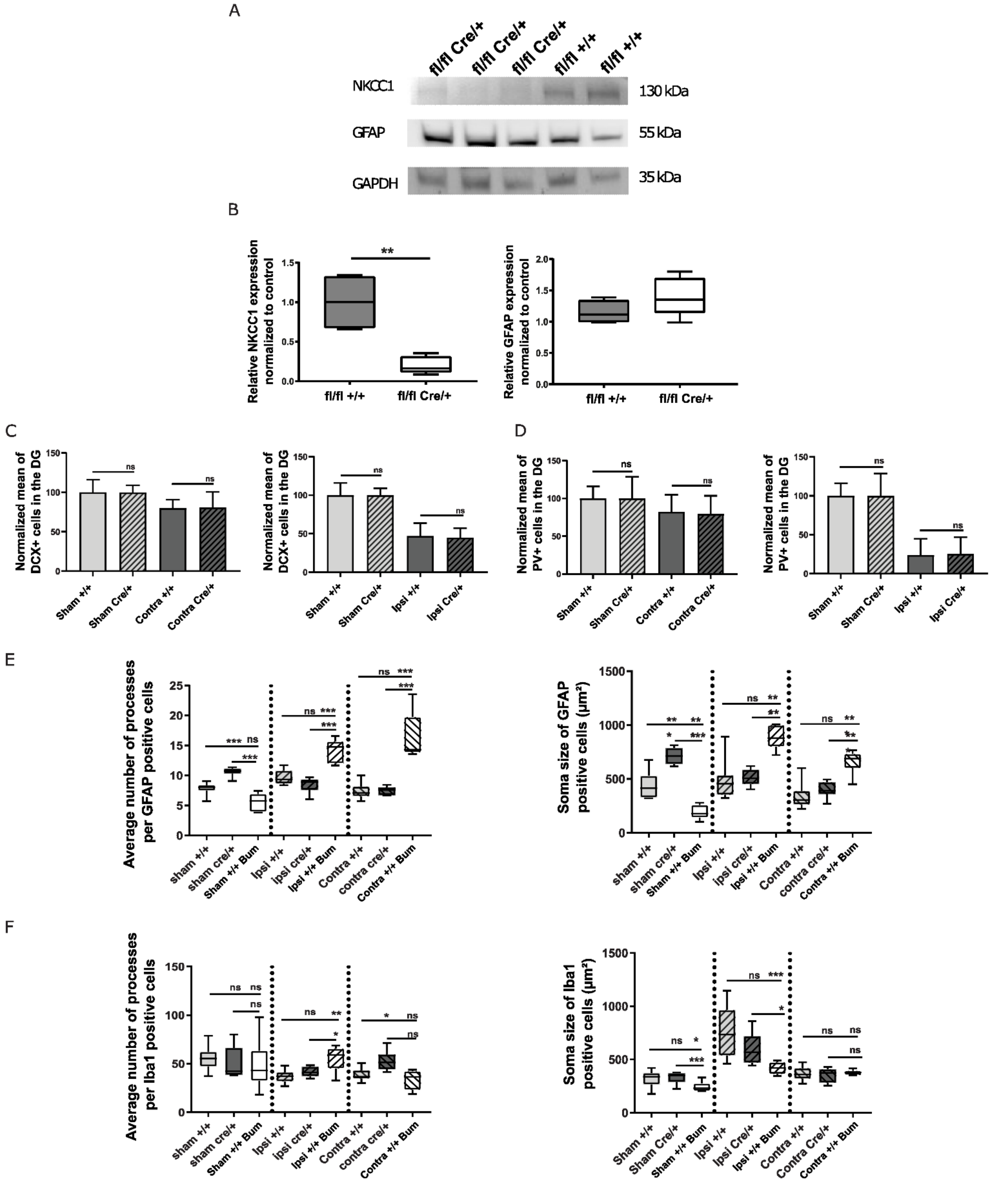

Supplementary Figure S2

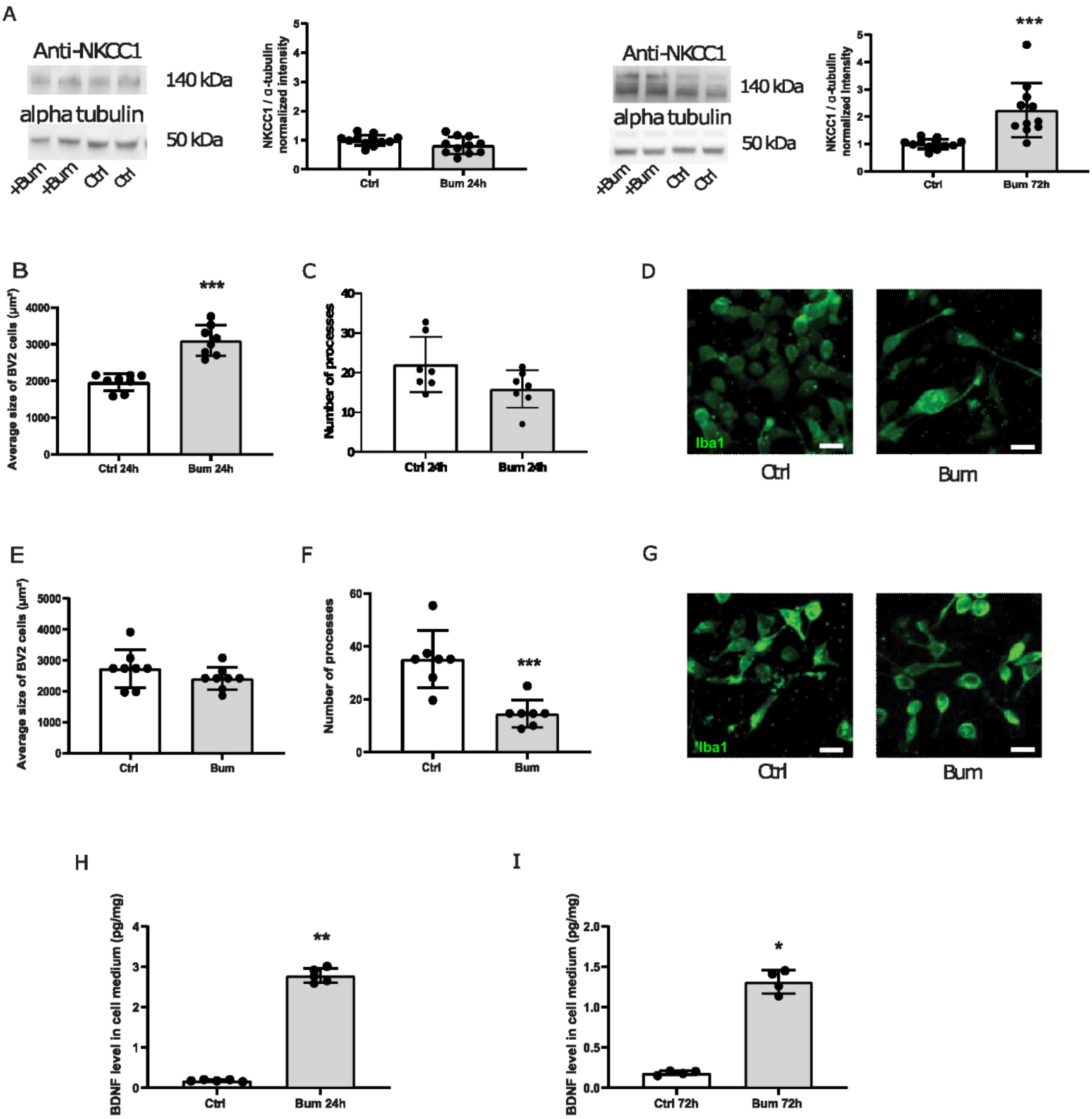

Supplementary Figure S3

Supplementary Figure S4

A

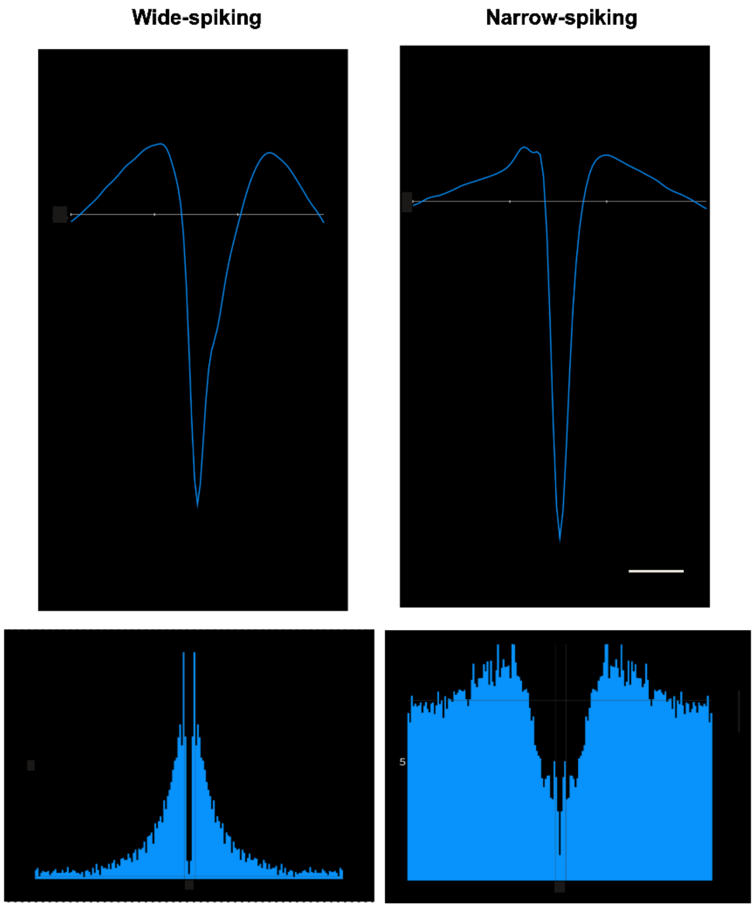
Wide-spiking
Narrow-spiking

B

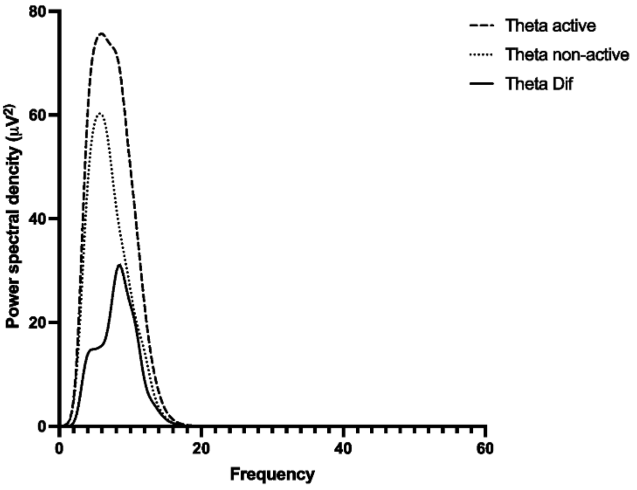
Theta active
Theta non-active
Theta Dif
Power spectral dencity (µV²)
0
20
40
60
80
Frequency

Figure	Panel	Normality	Test	Group compared	Mean Diff	SE of diff.	n1	n2	t /q / Z	DF	Significant	Adjusted p-value
1	B	No	Kruskal-Wallis	Sham vs. CCI Veh	13,1		11	13	2,67		*	0,023
				Sham vs. CCI Bum	2,476		11	17	0,5342		ns	>,999
				CCI Veh vs. CCI Bum	-10,63		13	17	2,408		*	0,048
	C	Yes	One-way ANOVA	Sham vs. CCI Veh	0,4912	0,1506	8	11	4,613	25	**	0,009
				Sham vs. CCI Bum	0,494	0,1575	8	9	4,436	25	*	0,012
				CCI Veh vs. CCI Bum	0,002778	0,1457	11	9	0,02697	25	ns	>,999
	D	Yes	One-way ANOVA	Sham vs. CCI Veh	0,5953	0,2231	7	6	3,773	17	*	0,041
				Sham vs. CCI Bum	0,4258	0,2144	7	7	2,809	17	ns	0,146
				CCI Veh vs. CCI Bum	-0,1695	0,2231	6	7	1,074	17	ns	0,732
	E	Yes	One-way ANOVA	Sham vs. CCI Veh	-36	8,119	3	5	6,271	10	**	0,003
				Sham vs. CCI Bum	-5	8,119	3	5	0,8709	10	ns	0,815
				CCI Veh vs. CCI Bum	31	7,031	5	5	6,235	10	**	0,003
	F	No	Two-way ANOVA	Centre								
				Sham vs. CCI Veh	-4	11,25	3	4	0,5029	27	ns	0,933
				Sham vs. CCI Bum	-1,4	10,76	3	5	0,1841	27	ns	0,991
				CCI Veh vs. CCI Bum	2,6	9,88	4	5	0,3722	27	ns	0,963
				Bras fermés								
				Sham vs. CCI Veh	0,3333	11,25	3	4	0,04191	27	ns	>,999
				Sham vs. CCI Bum	-11,27	10,76	3	5	1,481	27	ns	0,554
				CCI Veh vs. CCI Bum	-11,6	9,88	4	5	1,66	27	ns	0,478
				Bras ouverts								
				Sham vs. CCI Veh	-14,42	11,25	3	4	1,813	27	ns	0,417
				Sham vs. CCI Bum	7,333	10,76	3	5	0,9642	27	ns	0,776
				CCI Veh vs. CCI Bum	21,75	9,88	4	5	3,113	27	ns	0,089

	G	Yes	Two-way ANOVA	Centre								
				Sham vs. CCI Veh	16,18	17,85	5	5	1,282	34	ns	0,64
				Sham vs. CCI Bum	8,136	17,85	5	5	0,6447	34	ns	0,892
				CCI Veh vs. CCI Bum	-8,048	17,85	5	5	0,6377	34	ns	0,894
				Bras fermés								
				Sham vs. CCI Veh	-6,24	17,85	5	5	0,4944	34	ns	0,935
				Sham vs. CCI Bum	-30,54	17,85	5	5	2,42	34	ns	0,216
				CCI Veh vs. CCI Bum	-24,3	17,85	5	5	1,926	34	ns	0,372
				Bras ouverts								
				Sham vs. CCI Veh	-18,26	20,61	3	5	1,253	34	ns	0,653
				Sham vs. CCI Bum	10,46	20,61	3	5	0,7175	34	ns	0,868
				CCI Veh vs. CCI Bum	28,72	17,85	5	5	2,276	34	ns	0,256
	H	Yes	Two-way ANOVA	Sham vs. CCI Veh	4,306	0,5597	6	9	10,88	21	***	<,001
				Sham vs. CCI Bum	3,619	0,5597	6	9	9,144	21	***	<,001
				CCI Veh vs. CCI Bum	-0,6872	0,5006	9	9	1,941	21	ns	0,373
	I			Sham vs. CCI Veh	3,077	0,5016	6	9	8,676	21	***	<,001
				Sham vs. CCI Bum	0,355	0,5016	6	9	1,001	21	ns	0,762
				CCI Veh vs. CCI Bum	-2,722	0,4486	9	9	8,581	21	***	<,001
	J			Sham vs. CCI Veh	-0,5015	0,15	6	9	4,728	21	**	0,008
				Sham vs. CCI Bum	-0,3099	0,15	6	9	2,922	21	ns	0,121
				CCI Veh vs. CCI Bum	0,1916	0,1342	9	9	2,019	21	ns	0,345
	K	Yes	One-way ANOVA	Veh vs. Sham	-20,11	3,906	5	10	7,282	24	***	<,001
				Bum vs. Sham	-8,293	3,054	12	10	3,841	24	*	0,031
				Bum vs. Veh	11,82	3,796	12	5	4,404	24	*	0,013
	L	Yes	One-way ANOVA	Sham vs. Veh	-2,573	1,512	47	48	2,407	140	ns	0,208
				Sham vs. Bum	-0,4682	1,512	47	48	0,438	140	ns	0,9485

				Veh vs. Bum	2,105	1,504	48	48	1,98	140	ns	0,3438
2	B (left)	Yes	One-way ANOVA	Sham vs Contra Veh	0,5109	0,07071	15	15	7,226	27,57	***	< 0,001
				Sham vs. Contra Bum	0,2615	0,08104	15	15	3,227	27,44	**	0,01
				Contra Veh vs. Contra Bum	-0,2494	0,07707	15	15	3,236	26,18	**	0,01
	B (Right)	Yes	One-way ANOVA	Sham vs. Ipsi Veh	0,7786	0,07727	15	15	10,08	27,56	***	<,001
				Sham vs. Ipsi Bum	0,5347	0,06908	15	15	7,74	23,98	***	<,001
				Ipsi Veh vs. Ipsi Bum	-0,2439	0,06337	15	15	3,849	25,71	**	0,002
	D (left)	Yes	One-way ANOVA	Sham vs. Contra Veh	0,5147	0,08989	15	15	5,726	22,39	***	<,001
				Sham vs. Contra Bum	0,06503	0,12	15	15	0,5419	27,32	ns	0,994
				Sham vs. Contra Mino	-0,1637	0,1448	15	11	1,131	17,7	ns	0,826
				Contra Veh vs. Contra Bum	-0,4497	0,1018	15	15	4,42	20,4	**	0,002
				Contra Veh vs. Contra Mino	-0,6784	0,13	15	11	5,216	12,73	***	<,001
				Contra Bum vs. Contra Mino	-0,2287	0,1524	15	11	1,5	19,88	ns	0,59
	D (right)	Yes	One-way ANOVA	Sham vs. Ipsi Veh	0,9503	0,08458	15	15	11,24	18,88	***	<,001
				Sham vs. Ipsi Bum	0,3695	0,1024	15	15	3,608	27,33	**	0,007
				Sham vs. Ipsi Mino	0,3242	0,149	15	11	2,175	17,19	ns	0,22
				Ipsi Veh vs. Ipsi Bum	-0,5808	0,07426	15	15	7,822	20,51	***	<,001
				Ipsi Veh vs. Ipsi Mino	-0,6261	0,1313	15	11	4,768	11,36	**	0,003
				Ipsi Bum vs. Ipsi Mino	-0,04529	0,1434	15	11	0,3157	15,4	ns	>,999
	F	Yes	One-way ANOVA	Sham vs. CCI Veh	0,7517	0,0648	49	20	16,41	96	***	<,001
				Sham vs. CCI Bum	0,5171	0,06085	49	24	12,02	96	***	<,001
				Sham vs. CCI A	0,3359	0,09868	49	7	4,814	96	**	0,005
				CCI Veh vs. CCI Bum	-0,2346	0,07394	20	24	4,487	96	*	0,011
				CCI Veh vs. CCI A	-0,4158	0,1073	20	7	5,483	96	**	0,001
				CCI Bum vs. CCI A	-0,1812	0,1049	24	7	2,443	96	ns	0,315
	G	Yes	One-way ANOVA	Sham vs. CCI Veh	-1,089	0,07201	21	28	21,39	70	***	<,001
				Sham vs. CCI Bum	-0,4611	0,07454	21	24	8,748	70	***	<,001

				CCI Veh vs. CCI Bum	0,6283	0,06939	28	24	12,8	70	***	<,001
	I	Yes	One-way ANOVA	Sham vs. CCI Veh	0,2195	0,07344	50	20	2,989	98	*	0,018
				Sham vs. CCI Bum	-0,008475	0,06893	50	24	0,123	98	ns	>,999
				Sham vs. CCI A	-0,1285	0,1057	50	8	1,216	98	ns	0,618
				CCI Veh vs. CCI Bum	-0,228	0,08404	20	24	2,713	98	*	0,039
				CCI Veh vs. CCI A	-0,348	0,1161	20	8	2,997	98	*	0,018
				CCI Bum vs. CCI A	-0,12	0,1133	24	8	1,059	98	ns	0,715
	J	No	Kruskal-Wallis	Sham vs. CCI Veh contra	-26,61		20	28	4,533		***	<,001
				Sham vs. CCI Bum contra	-7,48		20	21	1,194		ns	0,697
				CCI Veh contra vs. CCI Bum contra	19,13		28	21	3,305		**	0,003
3	A	Yes	One-way ANOVA	ShNKCC1 + RFP vs. Tam + Bum	-78081	24835	5	5	4,446	16	*	0,029
				ShNKCC1 + RFP vs. Tam + RFP	10169	24835	5	5	0,5791	16	ns	0,976
				ShNKCC1 + RFP vs. Tam + ShNKCC1 + RFP	-99440	24835	5	5	5,663	16	**	0,005
				Tam + Bum vs. Tam + RFP	88250	24835	5	5	5,025	16	*	0,013
				Tam + Bum vs. Tam + ShNKCC1 + RFP	-21358	24835	5	5	1,216	16	ns	0,825
				Tam + RFP vs. Tam + ShNKCC1 + RFP	-109608	24835	5	5	6,242	16	**	0,002
	B	Yes	One-way ANOVA	ShNKCC1 + RFP vs. Tam + Bum	-18,64	6,253	11	13	4,215	44	*	0,023
				ShNKCC1 + RFP vs. Tam + RFP	8,636	6,509	11	11	1,877	44	ns	0,551
				ShNKCC1 + RFP vs. Tam + ShNKCC1 + RFP	-16,1	6,253	11	13	3,641	44	ns	0,062
				Tam + Bum vs. Tam + RFP	27,27	6,253	13	11	6,168	44	***	<,001
				Tam + Bum vs. Tam + ShNKCC1 + RFP	2,538	5,987	13	13	0,5996	44	ns	0,974
				Tam + RFP vs. Tam + ShNKCC1 + RFP	-24,73	6,253	11	13	5,594	44	**	0,002
4	A (left)	Yes	One-way ANOVA	Sham vs. CCI Veh	-2309	409,8	4	5	7,966	12	***	<,001
				Sham vs. CCI Bum	-3610	394,4	4	6	12,94	12	***	<,001
				CCI Veh vs. CCI Bum	-1301	370	5	6	4,975	12	*	0,011
	A (middle left)	Yes	One-way ANOVA	Sham vs. CCI Veh	17,11	129,1	7	8	0,1874	22	ns	0,99
				Sham vs. CCI Bum	-359,6	123	7	10	4,137	22	*	0,021

				CCI Veh vs. CCI Bum	-376,8	118,3	8	10	4,502	22	*	0,011
	A (middle right)	Yes	One-way ANOVA	Sham vs. CCI Veh	-60,02	22,77	7	8	3,728	23	*	0,038
				Sham vs. CCI Bum	-98,96	21,27	7	11	6,581	23	***	<,001
				CCI Veh vs. CCI Bum	-38,94	20,44	8	11	2,695	23	ns	0,16
	A (right)	Yes	One-way ANOVA	Sham vs. CCI Veh	-65,51	28,44	7	9	3,257	24	ns	0,075
				Sham vs. CCI Bum	-82,56	27,29	7	11	4,279	24	*	0,016
				CCI Veh vs. CCI Bum	-17,05	25,37	9	11	0,9506	24	ns	0,782
	C	Yes	One-way ANOVA	Sham Contra vs. CCI Veh contra	82,43	16,78	7	7	6,949	18	***	<,001
				Sham Contra vs. CCI Bum contra	22,86	16,78	7	7	1,927	18	ns	0,381
				CCI Veh contra vs. CCI Bum contra	-59,57	16,78	7	7	5,022	18	**	0,006
		Yes	One-way ANOVA	Sham ipsi vs. CCI Veh ipsi	96,82	11,78	7	6	11,62	17	***	<,001
				Sham ipsi vs. CCI Bum ipsi	36,37	11,32	7	7	4,544	17	*	0,013
				CCI Veh ipsi vs. CCI Bum ipsi	-60,45	11,78	6	7	7,255	17	***	<,001
	E	Yes	T-test	Bum M1 vs. Vehicle M1	8,703	2,053	6	5	4,239	9	**	0,002
				Bum M2 vs. Vehicle M2	0,876	5,836	6	5	0,1626	9	ns	0,874
	G	Yes	Brown-Forsythe ANOVA	Sham IL-4 vs. CCI Veh IL-4	-4,056	1,381	14	10	2,937	10,91	*	0,038
				Sham IL-4 vs. CCI Bum IL-4	-8,832	1,688	14	8	5,232	7,967	**	0,002
				CCI Veh IL-4 vs. CCI Bum IL-4	-4,776	2,096	10	8	2,279	14,32	ns	0,108
				Sham IL-10 vs. CCI Veh IL-10	0,5247	0,6465	14	4	0,8116	3,652	ns	0,805
				Sham IL-10 vs. CCI Bum IL-10	-1,016	1,723	14	4	0,5898	3,082	ns	0,906
				CCI Veh IL-10 vs. CCI Bum IL-10	-1,541	1,819	4	4	0,8473	3,762	ns	0,786
				Sham IL-6 vs. CCI Veh IL-6	-1,368	1,761	12	5	0,7767	4,182	ns	0,823
				Sham IL-6 vs. CCI Bum IL-6	-10,44	2,073	12	6	5,035	5,163	*	0,011
				CCI Veh IL-6 vs. CCI Bum IL-6	-9,069	2,695	5	6	3,365	8,974	*	0,023
5	A (left)	Yes	One-way ANOVA	Sham vs. CCI Veh	-460,9	275,2	8	8	2,368	21	ns	0,238
				Sham vs. CCI Bum	-625,9	275,2	8	8	3,216	21	ns	0,082
				CCI Veh vs. CCI Bum	-165	275,2	8	8	0,8478	21	ns	0,822

	A (middle left)	Yes	One-way ANOVA	Sham vs. CCI Veh	204,2	71,31	6	6	4,049	15	*	0,03
				Sham vs. CCI Bum	241,7	71,31	6	6	4,792	15	*	0,011
				CCI Veh vs. CCI Bum	37,5	71,31	6	6	0,7437	15	ns	0,86
	A (middle right)	Yes	One-way ANOVA	Sham vs. CCI Veh	-6,571	5,13	7	7	1,811	18	ns	0,424
				Sham vs. CCI Bum	-4,857	5,13	7	7	1,339	18	ns	0,619
				CCI Veh vs. CCI Bum	1,714	5,13	7	7	0,4725	18	ns	0,941
	A (right)	Yes	One-way ANOVA	Sham vs. CCI Veh	58,88	21,32	7	6	3,905	16	*	0,035
				Sham vs. CCI Bum	64,05	21,32	7	6	4,248	16	*	0,022
				CCI Veh vs. CCI Bum	5,167	22,13	6	6	0,3302	16	ns	0,97
	C	Yes	One-way ANOVA	Sham contra vs. CCI Veh contra	-1,4	10,3	6	6	0,1922	15	ns	0,99
				Sham contra vs. CCI Bum contra	13,6	10,3	6	6	1,867	15	ns	0,406
				CCI Veh contra vs. CCI Bum contra	15	10,3	6	6	2,059	15	ns	0,339
		Yes	One-way ANOVA	Sham ipsi vs. CCI Veh ipsi	32,97	6,613	6	6	7,05	15	***	<,001
				Sham ipsi vs. CCI Bum ipsi	15,77	6,613	6	6	3,373	15	ns	0,074
				CCI Veh ipsi vs. CCI Bum ipsi	-17,2	6,613	6	6	3,677	15	*	0,05
	E	Yes	T-test	Bum M1 vs Veh M1	0,03667	3,551	4	6	0,01033	8	ns	0,992
				Bum M2 vs Veh M2	11,9	4,188	5	6	2,841	9	*	0,019
	F	Yes	Brown-Forsythe ANOVA	Sham IL-4 vs. CCI Veh IL-4	0,3244	0,6112	10	4	0,5307	7,583	ns	0,931
				Sham IL-4 vs. CCI Bum IL-4	-0,4554	0,4277	10	4	1,065	11,31	ns	0,65
				CCI Veh IL-4 vs. CCI Bum IL-4	-0,7797	0,493	4	4	1,582	3,713	ns	0,412
				Sham IL-10 vs. CCI Veh IL-10	-0,8901	0,4169	9	4	2,135	6,911	ns	0,179
				Sham IL-10 vs. CCI Bum IL-10	-3,634	0,5035	9	4	7,217	5,28	**	0,002
				CCI Veh IL-10 vs. CCI Bum IL-10	-2,744	0,5418	4	4	5,064	5,588	**	0,006
				Sham IL-6 vs. CCI Veh IL-6	1,066	0,4951	10	4	2,153	11,75	ns	0,142
				Sham IL-6 vs. CCI Bum IL-6	1,043	0,6017	10	4	1,733	8,372	ns	0,299
				CCI Veh IL-6 vs. CCI Bum IL-6	-0,02318	0,5201	4	4	0,04456	5,054	ns	>,999
	G (left)	Yes	One-way ANOVA	Sham Veh vs. Sham Bum	-2,834	0,7918	4	3	5,061	9	*	0,025

				Sham Veh vs. CCI Veh	-1,834	0,7918	4	3	3,276	9	ns	0,165
				Sham Veh vs. CCI Bum	-11,41	0,7918	4	3	20,38	9	***	<,001
				Sham Bum vs. CCI Veh	0,9992	0,8464	3	3	1,669	9	ns	0,653
				Sham Bum vs. CCI Bum	-8,574	0,8464	3	3	14,33	9	***	<,001
				CCI Veh vs. CCI Bum	-9,573	0,8464	3	3	15,99	9	***	<,001
	G (right)	Yes	One-way ANOVA	Sham Veh vs. Sham Bum	-2,554	0,6462	3	3	5,591	8	*	0,018
				Sham Veh vs. CCi Veh	-1,267	0,6462	3	3	2,773	8	ns	0,278
				Sham Veh vs. CCI Bum	-4,591	0,6462	3	3	10,05	8	***	<,001
				Sham Bum vs. CCi Veh	1,287	0,6462	3	3	2,817	8	ns	0,267
				Sham Bum vs. CCI Bum	-2,037	0,6462	3	3	4,457	8	ns	0,054
				CCi Veh vs. CCI Bum	-3,324	0,6462	3	3	7,274	8	**	0,004
6	C	Yes	T-test	Day 1 Bum vs Veh	-9,147	3,408	12	18	2,68	28	**	0,004
		Yes	T-test	Day 5 Bum vs Veh	4,713	4,045	11	18	1,165	27	ns	0,338
	E	Yes	T-test	Day 1 Bum vs Veh	-0,07	0,016	47	54	4,32	99	***	<,001
		No	Mann Whitney	Day 5 Bum vs Veh	-0,055		31	48			**	0,001
7	A (left)	Yes	One-way ANOVA	Sham vs. Contra Veh	0,09915	0,1079	17	12	1,3	74	ns	0,94
				Sham vs. Contra Bum	0,4261	0,1079	17	12	5,585	74	**	0,002
				Contra Veh vs. Contra Bum	0,3269	0,1168	12	12	3,958	74	ns	0,069
	A (Right)	Yes	One-way ANOVA	Sham vs. Ipsi Veh	0,4624	0,1079	17	12	6,061	74	***	<,001
				Sham vs. Ipsi Bum	0,8954	0,114	17	10	11,1	74	***	<,001
				Ipsi Veh vs. Ipsi Bum	0,433	0,1225	12	10	4,998	74	**	0,009
S1	A (left)	Yes	One-way ANOVA	Sham vs, CCI Veh	-1.636	0.1499	32	40	15,44	109	***	<,001
				Sham vs, CCI Bum	-1.536	0.1500	32	40	14.49	109	***	<,002
				CCI Veh vs. CCI Bum	0.1006	0.1413	40	40	1,007	109	ns	.757
	A (right)	Yes	One-way ANOVA	Sham vs, CCI Veh	-0.078	0.03058	8	11	3.627	17	ns	.050
				Sham vs, CCI Bum	-0.059	0.03059	8	11	2.753	17	ns	.156

			CCI Veh vs. CCI Bum	-0,33	0.02803	11	11	0.9545	17	ns	.781
B (left)	Yes	One-way ANOVA	Sham vs, CCI Veh	-0.6844	0.09701	32	44	9.978	117	***	<,001
			Sham vs, CCI Bum	-0.5615	0.09701	32	44	8.186	117	***	<,001
			CCI Veh vs. CCI Bum	-0.1229	0.08902	44	44	1.953	117	ns	.354
B (right)	Yes	One-way ANOVA	Sham vs, CCI Veh	-0.04120	0.03488	8	12	1.671	29	ns	.474
			Sham vs, CCI Bum	-0,0121	0.03489	8	12	0.4905	29	ns	.936
			CCI Veh vs. CCI Bum	-0.05330	0.03120	12	12	2.416	29	ns	.219
C (left)	Yes	One-way ANOVA	Sham vs, CCI Veh	-1.022	0.08790	52	48	16.44	145	***	<,001
			Sham vs, CCI Bum	-0.8086	0.08790	52	48	13.01	145	***	<,001
			CCI Veh vs. CCI Bum	0.2129	0.08964	48	48	3.359	145	*	.049
C (right)	Yes	One-way ANOVA	Sham vs, CCI Veh	-0.1313	0.03825	11	11	4.856	28	**	.005
			Sham vs, CCI Bum	-0.1114	0.04031	11	9	3.907	28	*	.026
			CCI Veh vs. CCI Bum	0.01995	0.04031	11	9	0.6999	28	ns	.874
D (left)	Yes	One-way ANOVA	Sham vs, CCI Veh	-0.2370	0.06452	56	48	5.194	149	***	<,001
			Sham vs, CCI Bum	-0.2416	0.06453	56	48	5.297	149	***	<,001
			CCI Veh vs. CCI Bum	-0.004667	0.06695	48	48	0.09859	149	ns	.997
D (right)	Yes	One-way ANOVA	Sham vs, CCI Veh	-0.03578	0.02683	12	12	1.886	31	ns	.388
			Sham vs, CCI Bum	-0.07613	0.02814	12	10	3.826	31	*	.029
			CCI Veh vs. CCI Bum	-0.04035	0.02815	12	10	2.028	31	ns	.336
E (left)	Yes	One-way ANOVA	Sham vs, CCI Veh	-0.8872	0.1612	48	32	7.783	121	***	<,001
			Sham vs, CCI Bum	-1.135	0.1474	48	44	10.88	121	***	<,001
			CCI Veh vs. CCI Bum	-0.2476	0.1641	32	44	2.133	121	ns	.291
E (right)	Yes	One-way ANOVA	Sham vs, CCI Veh	-0.03711	0.04257	12	14	1.233	36	ns	.661
			Sham vs, CCI Bum	-0.07792	0.04332	12	13	2.544	36	ns	.184
			CCI Veh vs. CCI Bum	-0.04082	0.04168	14	13	1.385	36	ns	.595
F (left)	Yes	One-way ANOVA	Sham vs, CCI Veh	-1.073	0.1173	48	32	12,94	121	***	<,001

				Sham vs, CCI Bum	-0.5458	0.1073	48	44	7,193	121	***	<,001
				CCI Veh vs. CCI Bum	0.5277	0.1195	32	44	6,247	121	***	<,001
	F (right)	Yes	One-way ANOVA	Sham vs, CCI Veh	0.002423	0.04334	11	9	0.07908	30	ns	.998
				Sham vs, CCI Bum	-0.02389	0.03950	11	13	0.8552	30	ns	.819
				CCI Veh vs. CCI Bum	-0.02631	0.04181	9	13	0.8899	30	ns	.805
	G (left)	Yes	One-way ANOVA	Sham vs. CCI Veh	-1282	375,1	6	6	4,832	15	*	0,01
				Sham vs. CCI Bum	62,83	375,1	6	6	0,2369	15	ns	0,985
				CCI Veh vs. CCI Bum	1344	375,1	6	6	5,068	15	**	0,007
	G (middle)	Yes	One-way ANOVA	Sham vs. CCI Veh	301,1	90,52	4	5	4,704	11	*	0,017
				Sham vs. CCI Bum	-61,7	90,52	4	5	0,964	11	ns	0,779
				CCI Veh vs. CCI Bum	-362,8	85,34	5	5	6,012	11	**	0,004
	G (right)	Yes	One-way ANOVA	Sham vs. CCI Veh	-140,4	13,8	5	5	14,38	12	***	<,001
				Sham vs. CCI Bum	2,6	13,8	5	5	0,2664	12	ns	0,981
				CCI Veh vs. CCI Bum	143	13,8	5	5	14,65	12	***	<,001
S2	B (left)	No	Mann Whitney	fl/fl Cre/+ vs fl/fl +/+	0,8018		6	5			**	0,0095
	B (right)	No	Mann Whitney	fl/fl Cre/+ vs fl/fl +/+	0,391		6	4			ns	0,0571
	C (left)	Yes	T-test	Sham Cre/+ vs Sham +/+	0,4989	4,95	16	16	0,1008	30	ns	0,92
				Contra Cre/+ vs Contra +/+	0,5904	5,495	16	21	0,1074	35	ns	0,915
	C (right)	Yes	T-test	Sham Cre/+ vs Sham +/+	1,936	5,074	16	16	0,3816	30	ns	0,705
				Ipsi Cre/+ vs Ipsi +/+	2,219	5,238	16	16	0,4237	30	ns	0,675
	D (left)	Yes	T-test	Sham Cre/+ vs Sham +/+	1,562	9,989	9	16	0,1564	23	ns	0,877
				Contra Cre/+ vs Contra +/+	2,997	9,138	10	20	0,328	28	ns	0,745
	D (right)	Yes	T-test	Sham Cre/+ vs Sham +/+	2	9,697	9	16	0,2063	23	ns	0,838
				Ipsi Cre/+ vs Ipsi +/+	1,169	8,508	10	18	0,1374	23	ns	0,892
	E (left)	Yes	Brown-Forsythe ANOVA	sham +/+ vs. sham cre/+	-275,1	46,42	10	8	5,925	15,1	***	<,001
				sham +/+ vs. Sham Bum	245,1	43,9	10	10	5,584	14,06	***	<,001

				sham cre/+ vs. Sham Bum	520,2	33,69	8	10	15,44	14,45	***	<,001
				ipsi cre/+ vs. Ipsi +/+	18,57	62,85	8	11	0,2955	13,74	ns	0,987
				ipsi cre/+ vs. Ipsi Bum	-371,7	44,1	8	10	8,429	15,51	***	<,001
				Ipsi +/+ vs. Ipsi Bum	-390,3	67,36	11	10	5,794	16,49	***	<,001
				contra cre/+ vs. Contra +/+	63,58	39,97	7	13	1,591	16,35	ns	0,332
				contra cre/+ vs. Contra Bum	-260,7	45,72	7	10	5,701	14,94	***	<,001
				Contra +/+ vs. Contra Bum	-324,3	46,32	13	10	7,001	18,39	***	<,001
	E (right)	Yes	Brown-Forsythe ANOVA	sham +/+ vs. sham cre/+	-2,749	0,3149	18	8	8,73	14,3	***	<,001
				sham +/+ vs. Sham Bum	2,343	0,5125	18	10	4,571	11,66	**	0,002
				sham cre/+ vs. Sham Bum	5,092	0,5437	8	10	9,366	13,49	***	<,001
				ipsi cre/+ vs. Ipsi +/+	-1,309	0,5657	8	11	2,315	15,16	ns	0,098
				ipsi cre/+ vs. Ipsi Bum	-5,649	0,7616	8	10	7,418	15,18	***	<,001
				Ipsi +/+ vs. Ipsi Bum	-4,34	0,7257	11	10	5,98	14,62	***	<,001
				contra cre/+ vs. Contra +/+	0,1529	0,4117	7	13	0,3713	17,79	ns	0,975
				contra cre/+ vs. Contra Bum	-9,241	1,295	7	10	7,135	9,72	***	<,001
				Contra +/+ vs. Contra Bum	-9,394	1,31	13	10	7,17	10,17	***	<,001
	F (left)	Yes	Brown-Forsythe ANOVA	sham +/+ vs. sham cre/+	-275,1	46,42	10	8	5,925	15,1	***	<,001
				sham +/+ vs. Sham Bum	245,1	43,9	10	10	5,584	14,06	***	<,001
				sham cre/+ vs. Sham Bum	520,2	33,69	8	10	15,44	14,45	***	<,001
				ipsi cre/+ vs. Ipsi +/+	18,57	62,85	8	11	0,2955	13,74	ns	0,987
				ipsi cre/+ vs. Ipsi Bum	-371,7	44,1	8	10	8,429	15,51	***	<,001
				Ipsi +/+ vs. Ipsi Bum	-390,3	67,36	11	10	5,794	16,49	***	<,001
				contra cre/+ vs. Contra +/+	63,58	39,97	7	13	1,591	16,35	ns	0,332
				contra cre/+ vs. Contra Bum	-260,7	45,72	7	10	5,701	14,94	***	<,001
				Contra +/+ vs. Contra Bum	-324,3	46,32	13	10	7,001	18,39	***	<,001
	F (right)	Yes	Brown-Forsythe ANOVA	Sham +/+ vs. Sham Cre/+	-11,09	25,94	16	8	0,4275	18,12	ns	0,963
				Sham +/+ vs. Sham Bum	72,64	22,42	16	12	3,239	25,77	**	0,01

				Sham Cre/+ vs. Sham Bum	83,73	23,42	8	12	3,576	13,86	**	0,009
				Ipsi Cre/+ vs. Ipsi +/+	-153,3	81,36	8	13	1,884	18,91	ns	0,202
				Ipsi Cre/+ vs. Ipsi Bum	177,4	55,01	8	8	3,225	8,919	*	0,029
				Ipsi +/+ vs. Ipsi Bum	330,7	65,85	13	8	5,022	14,14	***	<,001
				Contra Cre/+ vs. Contra +/+	-13,84	33,86	6	12	0,4086	9,752	ns	0,967
				Contra Cre/+ vs. Contra Bum	-25,14	28,8	6	12	0,8731	5,589	ns	0,77
				Contra +/+ vs. Contra Bum	-11,31	20,21	12	12	0,5595	13,72	ns	0,922
S3	A (left)	Yes	T-test	Bum 24h vs Ctrl	0,1833	0,09981	12	11	1,836	21	ns	0,08
	A (right)	Yes	T-test	Bum 72h vs Ctrl	1,244	0,2907	12	11	4,278	21	***	<,001
	B	Yes	T-test	Bum 24h vs Ctrl	1136	167,7	8	8	6,771	14	***	<,001
	C	Yes	T-test	Bum 24h vs Ctrl	6,175	3,181	7	7	1,941	12	ns	0,076
	E	Yes	T-test	Bum 72h vs Ctrl	309,3	342	8	8	0,9044	14	ns	0,381
	F	Yes	T-test	Bum 72h vs Ctrl	22,89	4,844	7	7	4,727	12	***	<,001
	H	No	Mann-Whitney	Bum 24h vs Ctrl	2,579		5	5			**	0,008
	I	No	Mann-Whitney	Bum 72h vs Ctrl	1,145		4	4			*	0,029

Supplementary Table S1 | Descriptive statistics of all data sets.

Descriptive statistics of data sets per figure including the normality of the data sets, the test used, the groups compared, the difference between the group means, the SE of diff, the number of values per group, the DF, the statistically significant difference and the adjusted p-value.

SE of diff = Standard error of the difference between mean; DF = Degrees of freedom
